# *Shimia rhodophyticola* sp. nov., *Neomarinovum algicola* gen. nov., sp. nov., and *Phycorhabdus gracilariae* gen. nov., sp. nov., isolated from marine algae, and the taxonomic revision of nineteen taxa within the family *Roseobacteraceae*

**DOI:** 10.71150/jm.2607010

**Published:** 2026-10-31

**Authors:** Hui Seong Won, Hülya Bayburt, Byeong Jun Choi, Jeong Min Kim, Jae Kyeong Lee, Baolei Jia, Che Ok Jeon

**Affiliations:** 1 Department of Life Science, Chung-Ang University, Seoul 06974, Republic of Korea; 2 Xianghu Laboratory, Hangzhou 311231, PR China

**Keywords:** *Shimia rhodophyticola*, *Neomarinovum algicola*, *Phycorhabdus gracilariae*, new taxa, reclassification, *Roseobacteraceae*, marine algae

## Abstract

Three bacterial strains, W99^T^, S113^T^, and S268^T^, were isolated from marine algae and subjected to polyphasic taxonomic characterization. Phylogenetic and phylogenomic analyses placed W99^T^ within the genus *Shimia*, whereas S113^T^ and S268^T^ formed two distinct genus-level lineages within the family *Roseobacteraceae*. The three isolates were Gram-stain-negative, non-motile, and obligately aerobic, with optimum growth at 25, 25, and 30°C, respectively, and at 2.0% (w/v) NaCl. Ubiquinone-10 was the sole respiratory quinone in all three strains. The major fatty acids were 11-methyl C_18:1_ *ω*7*c* and summed feature 8 (C_18:1_ *ω*7*c* and/or C_18:1_ *ω*6*c*) in strain W99^T^; C_16:0_ and summed feature 8 in strain S113^T^; and 11-methyl C_18:1_ *ω*7*c*, cyclo-C_19:0_ *ω*8*c*, and summed feature 8 in strain S268^T^. The major polar lipids were phosphatidylglycerol and phosphatidylcholine, with diphosphatidylglycerol additionally detected in strain S268^T^. Polyphasic taxonomic analyses demonstrated that strain W99^T^ represents a novel species of the genus *Shimia*, for which the name *Shimia rhodophyticola* sp. nov. is proposed (W99^T^ = KACC 24154^T^ = JCM 38196^T^), whereas strains S113^T^ and S268^T^ represent two novel genera and species, *Neomarinovum algicola* gen. nov., sp. nov. (S113^T^ = KACC 23417^T^ = JCM 38210^T^) and *Phycorhabdus gracilariae* gen. nov., sp. nov. (S268^T^ = KACC 24156^T^ = JCM 38211^T^), respectively. In addition, comprehensive phylogenomic and genome-based relatedness analyses support the taxonomic revision of nineteen taxa within the family *Roseobacteraceae*, providing a revised genome-based taxonomic framework that better reflects the evolutionary relationships within the family.

## Introduction

The family *Roseobacteraceae* Liang et al. 2021, recently separated from the family *Rhodobacteraceae* based on genome-scale phylogenetic and genotypic analyses, comprises a diverse group of marine bacteria with broad physiological and ecological diversity (Liang et al., 2021). As of July 2026, the family includes 144 validly published genera isolated from a wide range of marine habitats, including seawater (Hwang and Cho, 2008; Lanclos et al., 2025), tidal mudflats (Lee et al., 2020), marine solar salterns (Wang et al., 2023), sea sand (Thongphrom et al., 2017), phycospheres (Coe et al., 2023), and marine algae (Jung et al., 2019; Kim et al., 2025; Lee et al., 2024). Recent genome-based studies have substantially improved our understanding of evolutionary relationships within the family (Wirth and Whitman, 2018; Zhang et al., 2023), but the current classification of several genera remains incongruent with their phylogenomic placement.

The genus *Shimia* was established with *Shimia marina* as the type species (Choi and Cho, 2006) was subsequently expanded through taxonomic reclassifications, including the transfer of *Thalassobius aestuarii* (Yi and Chun, 2006) and *Thalassobius abyssi* (Nogi et al., 2016) to the genus *Shimia* by Wirth and Whitman (2018). The genus currently comprises 13 species with validly published names and one species whose name has not been validly published. Members of the genus have been recovered from diverse marine environments, including seawater (Arahal et al., 2019; Chen et al., 2023; Hameed et al., 2013; Kim et al., 2008), sediments (Nogi et al., 2015, 2016; Yi and Chun, 2006; Zhu et al., 2021), corals (Chen et al., 2011), and marine invertebrates (Hyun et al., 2013; Kim et al., 2014). They are characterized as Gram-stain-negative, aerobic, rod-shaped bacteria possessing ubiquinone-10 (Q-10) as the predominant respiratory quinone, with 11-methyl C_18:1_ *ω*7*c* and summed feature 8 (C_18:1_ *ω*7*c* and/or C_18:1_ *ω*6*c*) as the major fatty acids. The major polar lipids are typically phosphatidylglycerol (PG) and phosphatidylcholine (PC), whereas phosphatidylethanolamine (PE) and diphosphatidylglycerol (DPG) vary among species. However, recent genome-based studies have suggested that the current circumscription of *Shimia*, as well as several other genera within the family *Roseobacteraceae*, does not fully reflect their evolutionary relationships.

Three isolates, W99^T^, S113^T^, and S268^T^, were recovered from marine algal phycospheres during our investigation of algae-associated bacterial diversity (Bayburt et al., 2024, 2026; Kim et al., 2025). Their taxonomic positions were evaluated using phylogenetic, genomic, phenotypic, and chemotaxonomic evidence. Genome-scale comparisons of selected *Roseobacteraceae* taxa additionally identified several discrepancies between their current classifications and inferred evolutionary relationships. Accordingly, this study describes *Shimia rhodophyticola* sp. nov., *Neomarinovum algicola* gen. nov., sp. nov., and *Phycorhabdus gracilariae* gen. nov., sp. nov., and proposes the taxonomic revision of nineteen taxa within the family *Roseobacteraceae*. These taxonomic revisions provide a revised genome-based framework that better reflects the evolutionary relationships within the family *Roseobacteraceae*.

## Materials and Methods

### Bacterial isolation and cultivation

Strains W99^T^, S113^T^, and S268^T^ were isolated from the phycospheres of marine algae collected from the Republic of Korea as previously described (Kim et al., 2025). Strain W99^T^ was isolated from the marine red alga *Schizymenia* sp. collected at Guryepo Beach (36°53′30.6″ N, 126°11′52.8″ E), strain S113^T^ from the marine green alga *Chaetomorpha* sp. collected at Nogu Port (34°45′17.0″ N, 128°03′20.9″ E), and strain S268^T^ from the marine red alga *Gracilaria* sp. collected at Mulgeon Port (34°46′50.5″ N, 128°02′56.5″ E). In brief, loosely associated microorganisms were removed from the algal samples by washing with sterile artificial seawater (ASW; 20.0 g NaCl, 2.9 g MgSO_4_, 4.5 g MgCl_2_·6H_2_O, 0.6 g KCl, 1.8 g CaCl_2_·2H_2_O per liter). The washed samples were homogenized with a T10 basic homogenizer (IKA, Germany), and serial dilutions of the homogenates were prepared in sterile ASW. Each dilution (100 µl) was inoculated onto marine agar (MA; MBcell, Korea) and incubated aerobically at 25°C until colony formation. Colony differentiation was performed by restriction analysis of 16S rRNA gene amplicons. The genes were amplified with primers 27F and 1492R (Lane, 1991), followed by digestion of the PCR products with HaeIII and HhaI. Restriction profiles were resolved on 2% agarose gels, and amplicons representing distinct profiles were selected for sequencing with primer 340F (Kim et al., 2025). Sequencing was performed by Macrogen (Korea), and the resulting sequences were compared with those of type strains of validly published species using the EzBioCloud Identify service (Chalita et al., 2024). Based on these analyses, strains W99^T^, S113^T^, and S268^T^ were selected for further taxonomic characterization. Routine cultivation was performed aerobically on MA or in marine broth (MB; MBcell) at 25°C for 3 days. For long-term preservation, cultures were stored at −80°C in MB supplemented with 15% (v/v) glycerol.

### Phylogenetic analysis using 16S rRNA gene sequences

Nearly complete 16S rRNA gene sequences of strains W99^T^, S113^T^, and S268^T^ were generated from PCR products amplified with primers 27F and 1492R. Additional sequence reads were obtained with primers 518R (5′-ATT ACC GCG GCT GCT GG-3′) and 805F (5′-GAT TAG ATA CCC TGG TAG TC-3′) (Kim et al., 2025), and the reads generated with 340F, 518R, and 805F were assembled to obtain the final sequences. Sequence similarities between the isolates and their closest type strains were determined using the EzBioCloud server (Chalita et al., 2024). For phylogenetic reconstruction, the 16S rRNA gene sequences were aligned with Infernal v1.1.4 using the Rfam RF00177 covariance model (Nawrocki and Eddy, 2013). Maximum-likelihood (ML), neighbor-joining (NJ), and maximum-parsimony (MP) analyses were independently conducted in MEGA11 (Tamura et al., 2021), and node support was assessed from 1,000 bootstrap replicates. Based on 16S rRNA gene sequence similarities and phylogenomic relationships, *Shimia aestuarii* KCTC 12049^T^, *Thiosulfatihalobacter marinus* KCTC 82723^T^, *Marinovum algicola* JCM 21266^T^, and *Primorskyibacter aestuariivivens* KCTC 52455^T^ were selected as reference strains for comparative genomic, phenotypic, and chemotaxonomic analyses. The reference strains for phenotypic and chemotaxonomic comparisons were selected primarily based on their taxonomic relevance to strains W99^T^ and S113^T^. For strain S268^T^, closely related taxa were included in the phylogenomic and genome-relatedness analyses to assess its taxonomic position, whereas additional strains were not included in the experimental phenotypic and chemotaxonomic comparisons because these analyses were intended to provide supplementary characterization rather than to determine its genus-level affiliation.

### Ecological distribution analysis based on 16S rRNA gene sequences

To estimate the ecological distribution of strains W99^T^, S113^T^, and S268^T^, their 16S rRNA gene sequences were queried against 296,393 metagenomic 16S rRNA amplicon datasets representing diverse environments using the Integrated Microbial Next-Generation Sequencing (IMNGS) platform (Lagkouvardos et al., 2016) with a 99.0% sequence similarity threshold. The datasets included marine and freshwater environments (seawater, estuaries, marine sediments, and freshwater), marine hosts (algae, corals, sponges, crustaceans, molluscs, oysters, and sea anemones), beach sand, biofilms, and terrestrial and engineered environments, including soil, compost, plants, wastewater, and activated sludge.

### Whole genome sequencing, genome-based phylogeny, and genome-relatedness analysis

Genomic DNA was extracted from strains W99^T^, S113^T^, and S268^T^ using the Wizard Genomic DNA Purification Kit (Promega, USA) according to the manufacturer’s instructions. Libraries were prepared using the Ligation Sequencing Kit (SQK-LSK109) with Native Barcoding Expansions EXP-NBD104 and EXP-NBD114 (Oxford Nanopore Technologies, UK) and sequenced on a MinION platform using R9.4.1 chemistry. Basecalling was performed in MinKNOW using the corresponding R9.4.1 MinION model. Reads were adapter-trimmed with Porechop v0.2.4 and filtered with NanoFilt v2.8.0 using minimum thresholds of Q10 and 200 bp. De novo assembly of the filtered reads was performed with Flye v2.9.1 in nano-hq mode (Kolmogorov et al., 2019), specifying a 5 Mb genome size and 50× coverage. The assemblies underwent one Flye polishing cycle, with no subsequent polishing step. Mean genome coverage was obtained from the final Flye assembly statistics. Genome completeness and contamination were assessed using CheckM2 v1.0.2 (Chklovski et al., 2023), and genomes were annotated using the NCBI Prokaryotic Genome Annotation Pipeline (PGAP; software revision 6.11).

Phylogenomic analysis included 206 genomes representing members of the family *Roseobacteraceae*, selected based on the phylogenomic relationships reported by Liang et al. (2021), with emphasis on taxa closely related to the three isolates and those relevant to the proposed taxonomic revisions. Additional closely related genera were selected based on 16S rRNA gene sequence similarities, and all available type-strain genomes of validly published species within the selected genera were included. GTDB-Tk v2.4.1 with GTDB release 226 (Chaumeil et al., 2022) was used in the de novo workflow to identify and align the bac120 single-copy marker proteins and to generate the trimmed concatenated alignment. The canonical bacterial mask was applied, with missing markers represented as gaps. The resulting concatenated alignment was used to reconstruct an ML phylogenomic tree in MEGA11 (Tamura et al., 2021) with 1,000 bootstrap replicates.

Genome relatedness was quantified using OrthoANIu for average nucleotide identity (ANI) (Yoon et al., 2017), Genome-to-Genome Distance Calculator (GGDC 3.0) with formula 2 for digital DNA-DNA hybridization (dDDH) (Meier-Kolthoff et al., 2022), and EzAAI v1.2.3 for average amino acid identity (AAI) (Kim et al., 2021). Percentage of conserved proteins (POCP) values were obtained according to Qin et al. (2014). AAI matrices were hierarchically clustered and visualized with ComplexHeatmap v2.14.0 in R v4.2.3, whereas pairwise ANI, AAI, and POCP relationships among members of the family *Roseobacteraceae* were plotted using ggplot2 and ggpubr.

### Phenotypic, physiological, and biochemical characterization

Physiological growth limits of strains W99^T^, S113^T^, and S268^T^ were determined using MA or MB, as appropriate. Temperature tolerance was assessed on MA from 5 to 50°C at 5°C intervals, whereas pH tolerance was examined in MB after 3 days of incubation over the range pH 3.0–12.0 at 1.0-unit intervals using citrate (pH 3.0–5.0), phosphate (pH 6.0–8.0), or carbonate-bicarbonate (pH 9.0–12.0) buffers. NaCl tolerance was evaluated in laboratory-prepared MB containing 0–10°C (w/v) NaCl. Growth on different nutrient media was tested on MA, R2A agar, LB agar, TSA, and NA adjusted to approximately 2% (w/v) NaCl after incubation at 25°C for 3 days. Unless otherwise specified, subsequent characterization was performed at the optimum temperature of each strain (25°C for strains W99^T^ and S113^T^ and 30°C for strain S268^T^). The ability to grow anaerobically was tested on MA for 21 days at each strain’s optimum temperature using a GasPak Plus system (BBL, USA). Morphological characteristics and motility were observed by phase-contrast microscopy (Zeiss Axio Scope.A1; Carl Zeiss, Germany) using cells grown on MA for 3 days at the optimum temperature of each strain. Cell ultrastructure was further examined by transmission electron microscopy (JEM-F200; JEOL, Japan) after negative staining with UranyLess (Electron Microscopy Sciences, USA) for 1 min (Benmeradi et al., 2015). Gliding behavior was examined on MA solidified with 0.3% (w/v) agar following Bernardet et al. (2002), and Gram reactions were determined with a commercial staining kit (bioMérieux, France). Catalase and oxidase activities were determined by bubble production in 3% (v/v) H_2_O_2_ (Junsei, Japan) and oxidation of 1% (w/v) tetramethyl-*p*-phenylenediamine (Merck, USA), respectively (Smibert and Krieg, 1994). All reference strains were examined under the same experimental conditions as the three isolates to permit direct phenotypic comparison. Substrate-degrading activities toward esculin, casein, starch, tyrosine, Tween 20, and Tween 80 were examined on MA according to Smibert and Krieg (1994). Additional biochemical characteristics were determined using the API 20NE system (bioMérieux, France) with inocula prepared in ASW according to the manufacturer's instructions.

### Chemotaxonomic characterization

Chemotaxonomic analyses were conducted with exponentially growing cultures. Quinones prepared from W99^T^, S113^T^, and S268^T^ according to Minnikin et al. (1984) were separated by reversed-phase HPLC using an LC-20A system equipped with an SPD-M20A diode-array detector and a Kromasil column (250 × 4.6 mm). Methanol/isopropanol (2:1, v/v) served as the mobile phase at 1 ml/min. Fatty acid profiles of the isolates and reference strains were determined from exponential-phase MB cultures (OD_600_ = 0.7–0.8) grown at their respective optimum temperatures. Fatty acid methyl esters prepared by the standard MIDI procedure were analyzed by gas chromatography (Hewlett Packard 6890), and components were assigned using the RTSBA6 database of Sherlock MIS v6.0B (Sasser, 1990). Polar lipid extracts prepared from exponentially growing cultures were resolved by two-dimensional thin-layer chromatography (TLC) following Minnikin et al. (1984). Total polar lipids, aminolipids, phospholipids, and glycolipids were detected with 10% (w/v) ethanolic molybdophosphoric acid, ninhydrin, Dittmer-Lester reagent, and *α*-naphthol/sulfuric acid, respectively; authentic PG, PC, and DPG standards were used for confirmation.

## Results and Discussion

### Isolation and phylogenetic characteristics based on 16S rRNA gene sequences

Screening of bacteria recovered from marine algal phycospheres yielded three putatively novel *Roseobacteraceae* isolates, designated W99^T^, S113^T^, and S268^T^. Their nearly complete 16S rRNA gene sequences were 1,404, 1,435, and 1,403 bp, respectively. Strain W99^T^ shared the highest 16S rRNA gene sequence similarity with *S. aestuarii* DSM 15283^T^ (99.9%), followed by *Shimia abyssi* JAMH 043^T^ (98.3%). Strain S113^T^ was most closely related to *Rhodalgimonas zhirmunskyi* KMM 6723^T^ (97.3%) and *Shimia sediminis* ZQ172^T^ (97.2%), whereas strain S268^T^ shared the highest sequence similarity with *S. aestuarii* DSM 15283^T^ (96.2%) and *Aestuariibius insulae* DBTF-13^T^ (96.2%). Based on the accepted 16S rRNA gene sequence similarity threshold for species delineation (98.5–98.7%) (Riesco and Trujillo, 2024), strains S113^T^ and S268^T^ are likely to represent novel species, whereas the taxonomic status of strain W99^T^ could not be resolved based solely on 16S rRNA gene sequence analysis.

An NJ phylogenetic tree based on 16S rRNA gene sequences showed that strain W99^T^ formed a robust cluster with *S. aestuarii* DSM 15283^T^ (100% bootstrap support; Fig. 1). However, this clade, together with several other *Shimia* species, was clearly separated from the lineage containing the type species, *Shimia marina*. This topology was consistently recovered in the ML and MP trees (Fig. S1), indicating that 16S rRNA gene sequence-based phylogenies provide insufficient resolution to accurately resolve phylogenetic relationships within the genus *Shimia*. Strain S113^T^ consistently clustered with *Phycobium rhodophyticola* D3-12^T^ in the NJ, ML, and MP trees, but the bootstrap support was low in all cases, indicating that its phylogenetic position could not be resolved confidently. In contrast, strain S268^T^ formed a distinct phylogenetic lineage in the NJ, ML, and MP trees, but its phylogenetic position relative to neighboring taxa remained unresolved. Collectively, these results demonstrate that the genus-level affiliations of strains W99^T^, S113^T^, and S268^T^ could not be determined confidently based solely on 16S rRNA gene phylogeny, necessitating genome-based analyses to clarify their taxonomic positions.

Furthermore, the 16S rRNA gene phylogenies showed that several genera within the family *Roseobacteraceae* did not form distinct monophyletic clades but were interspersed throughout the trees. This pattern indicates that 16S rRNA gene sequences provide insufficient phylogenetic resolution for robust genus-level classification within the family and suggests that the taxonomy of several taxa should be reassessed using genome-based approaches.

### Ecological distribution

Ecological distribution analysis using the IMNGS platform at a 99.0% 16S rRNA gene sequence similarity threshold showed that sequences closely related to strains W99^T^, S113^T^, and S268^T^ were predominantly detected in marine environments (Table S1). Sequences closely related to W99^T^ and S113^T^ were frequently detected in sea anemone microbiomes (76.7% prevalence; average relative abundance [ARA], 3.08%) and the marine diatom *Seminavis robusta* (44.4% and 88.9% prevalence, respectively). Closely related sequences were also detected in various marine invertebrate-associated datasets, including those from the sea urchin *Tripneustes gratilla*, spiny lobster *Panulirus ornatus*, crab *Cancer borealis*, and corals, whereas sequences related to S268^T^ generally showed lower prevalence. In contrast, sequences related to the three strains were rare or absent in terrestrial and anthropogenic environments, including compost, plants, and human-associated samples. Although the strains were isolated from marine algal phycospheres, the detection of closely related sequences across diverse marine host-associated habitats suggests that phylogenetically related bacteria may occur beyond algal hosts. Overall, these results suggest a predominantly marine distribution of 16S rRNA gene sequences closely related to the three strains.

### Genome sequencing and genomic features

After quality filtering, read yields for strains W99^T^, S113^T^, and S268^T^ were 335.8, 323.7, and 640.9 Mb, respectively, with corresponding read N50 values of 19,422, 11,491, and 28,075 bp. De novo assembly generated draft genomes of approximately 4,262, 4,314, and 3,864 kb, with average coverage depths of 76.0×, 73.0×, and 162.0× and assembly N50 values of 4,008,202, 4,022,761, and 3,667,163 bp, respectively. The genome-derived 16S rRNA gene sequences were identical to those obtained by PCR-based sequencing. CheckM2 indicated high estimated completeness and low estimated contamination for all three assemblies, with completeness and contamination values of 100.0% and 0.6% for W99^T^, 100.0% and 0.2% for S113^T^, and 93.2% and 1.2% for S268^T^, respectively (Chklovski et al., 2023).

The genomic DNA G + C contents of strains W99^T^, S113^T^, and S268^T^ were 60.5, 60.0, and 56.1 mol%, respectively, consistent with those of their closely related taxa. Detailed genomic features, including genome size, G + C content, and numbers of coding sequences, tRNA genes, and rRNA genes, are summarized in Table S2.

### Genome-based phylogeny and genome relatedness

An ML phylogenomic tree based on the concatenated amino acid sequences of 120 conserved bacterial marker genes placed strain W99^T^ within the genus *Shimia*, where it formed a robust clade with *S. aestuarii* DSM 15283^T^ (99% bootstrap support) within the lineage containing the type species, *Shimia marina* (Fig. 2). This topology contrasted with the 16S rRNA gene phylogeny, in which strain W99^T^ was separated from the type-species lineage. Notably, species currently assigned to *Shimia* did not form a monophyletic clade in the phylogenomic tree, supporting the need for taxonomic revision of the genus. In contrast, strains S113^T^ and S268^T^ each formed an independent lineage distinct from their closest relatives. Strain S113^T^ was clearly separated from *Marinovum algicola* FF3^T^, *Marinovum sedimenti* KMM 9989^T^, and *Primorskyibacter aestuariivivens* OITF-36^T^, whereas strain S268^T^ formed a distinct lineage separate from *Parasulfitobacter algicola* 1151^T^, *Pseudaestuariivita rosea* H15^T^, and *Algirhabdus cladophorae* KMM 6494^T^. These results support the placement of strain W99^T^ within the genus *Shimia* and indicate that strains S113^T^ and S268^T^ represent two novel genus-level lineages within the family *Roseobacteraceae*.

The ANI and dDDH values between strain W99^T^ and its closest type strain, *S. aestuarii* DSM 15283^T^, were 94.9% and 59.6%, respectively, whereas those between strain W99^T^ and all other related type strains were ≤ 79.4% ANI and ≤ 21.8% dDDH (Table S3). Although the ANI value of 94.9% falls within the borderline range for species delineation (94–96%), the dDDH value of 59.6% is below the 70% species threshold (Riesco and Trujillo, 2024), supporting the recognition of strain W99^T^ as a novel species of the genus *Shimia*. Strains S113^T^ and S268^T^ also exhibited low ANI and dDDH values relative to their closest type strains, with maximum values of 73.2% ANI and 24.9% dDDH for strain S113^T^ and 71.0% ANI and 20.7% dDDH for strain S268^T^ (Table S3). These values indicate substantial genomic divergence from currently recognized taxa. However, because universally accepted ANI and dDDH thresholds for genus delineation have not been established, these indices alone are insufficient to justify recognition of novel genera. Furthermore, clustering patterns based on ANI and dDDH as well as AAI (Fig. S2, Table S3) were not fully congruent with the phylogenomic topology (Fig. 2), indicating that genome-relatedness indices and phylogenomic analyses capture different aspects of evolutionary relationships. Together, these findings suggest that taxonomic assignments within the family *Roseobacteraceae* should be based on multiple complementary genomic criteria, with genome-relatedness indices interpreted in conjunction with phylogenomic evidence rather than used as standalone measures.

Several genome-based metrics have been proposed for genus delineation, among which AAI is one of the most widely used. However, no universally accepted AAI threshold for genus delineation has been established. Konstantinidis et al. (2017) reported that AAI values generally range from 65–95% among members of the same genus and 45–65% among members of the same family, whereas Luo et al. (2014) found that AAI values between members of different genera typically range from 60% to 80% and rarely exceed 85%, indicating substantial overlap among taxonomic groups. More recently, Nicholson et al. (2020) proposed that AAI values ≥ 76% support assignment to the same genus, whereas values ≤ 74% indicate different genera. Pairwise AAI analyses showed that strains S113^T^ and S268^T^ shared maximum AAI values of only 71.8% and 70.8%, respectively, with their closest type strains (Fig. S2). These values fall below the genus boundary proposed by Nicholson et al. (2020) and, together with their distinct phylogenomic positions, support the recognition of strains S113^T^ and S268^T^ as the type strains of two novel genera within the family *Roseobacteraceae*.

Beyond the taxonomic positions of the novel isolates, the phylogenomic analysis also revealed inconsistencies in the classification of several currently recognized taxa within the family *Roseobacteraceae*, particularly members of the genus *Shimia*. In the phylogenomic tree, species assigned to *Shimia* did not form a monophyletic clade but were divided into multiple lineages, with members of the genus *Cognatishimia* nested between *Shimia* clades (Fig. 2). Consistent with this topology, *Cognatishimia* species shared low AAI values (71.0–71.8%) with the type species, *S. marina* DSM 26895^T^, confirming their clear separation from the *Shimia* type-species lineage (Fig. S2A). These results indicate that the current circumscription of *Shimia* does not accurately reflect its phylogenomic and evolutionary relationships.

Species comprising the *Shimia* lineage lacking the type species, including '*Shimia fortis*' SDUM112013 (Dong et al., 2026), *Shimia thalassica* CECT 7735^T^ (Arahal et al., 2019), *Shimia gijangensis* DSM 100564^T^ (Arahal et al., 2019), *Shimia sediminis* ZQ172^T^ (Zhu et al., 2021), and *Shimia litoralis* CL-ES2^T^ (Zhang et al., 2023), shared AAI values of only 72.9–73.7% with *S. marina* DSM 26895^T^, indicating substantial genomic divergence from the type-species lineage (Fig. S2A). Likewise, *Shimia biformata* JCM 18818^T^ formed an independent phylogenomic lineage and shared an AAI value of only 72.5% with *S. marina* DSM 26895^T^, whereas *Shimia ponticola* WX04^T^ also formed an independent lineage and shared AAI values of ≤ 68.7% with all neighboring taxa (Fig. S2A). Together with the phylogenomic evidence, these genomic characteristics support the reclassification of the type-species-lacking *Shimia* lineage, *S. biformata*, and *S. ponticola* into three separate novel genera distinct from the *Shimia* type-species lineage within the family *Roseobacteraceae*.

In contrast, *Thiosulfatihalobacter marinus* GL-11-2^T^, the sole validly published species of the genus *Thiosulfatihalobacter* (Wu et al., 2022), was nested within the *Shimia* sensu stricto clade containing the type species, *S. marina* DSM 26895^T^ (Fig. 2). Consistent with this phylogenomic placement, *Thiosulfatihalobacter marinus* shared an AAI value of 75.6% with *Shimia marina* DSM 26895^T^ (Fig. S2A). Although *T. marinus* shared an AAI value of 75.6% with *S. marina* DSM 26895^T^, which falls within the intermediate range for genus delineation (Fig. S2A), its placement within the *Shimia* type-species lineage provides the primary evidence for its transfer to *Shimia*, with the AAI value serving as complementary evidence.

Beyond the taxonomic revisions involving *Shimia* and *Thiosulfatihalobacter*, the phylogenomic analysis also revealed inconsistencies in the classification of several additional members of the family *Roseobacteraceae*, including twelve species with validly published names (Figs. 2 and S2). Among them, *Ruegeria discodermiae* 2205SS24-7^T^, *Ruegeria jejuensis* 2304DJ69-11^T^, *Ruegeria spongiae* 2012CJ41-6^T^, and *Ruegeria hyattellae* 2012CJ15-1^T^ formed a well-supported monophyletic clade distinct from the *Ruegeria sensu* stricto clade containing the type species, *Ruegeria atlantica* CECT 4292^T^ (Fig. 2). Instead, these four species clustered with members of the genus *Falsiruegeria*. They shared high AAI values among themselves (88.5–93.8%) but only 75.6–75.9% AAI with *R. atlantica* CECT 4292^T^ and 75.0–75.5% with the type species of *Falsiruegeria*, *Falsiruegeria mediterranea* CECT 7615^T^ (Fig. S2A). Although these AAI values fall within the intermediate range for genus delineation proposed by Nicholson et al. (2020), the formation of a well-supported and cohesive phylogenomic lineage distinct from the type-species lineages of both *Ruegeria* and *Falsiruegeria* provides the primary evidence for recognizing these four species as a separate genus, while their high within-lineage AAI values provide additional genomic support. Although 16S rRNA gene phylogeny has limited resolution for genus-level classification, it also supports their separation from *Falsiruegeria* (Figs. 1 and S1). Collectively, these findings support the reclassification of the four species into a novel genus within the family *Roseobacteraceae*.

Likewise, *Lutimaribacter marinistellae* KCTC 42911^T^ formed an independent lineage clearly separated from the remaining members of the genus *Lutimaribacter* (Fig. 2). Although *Lutimaribacter marinistellae* was positioned closest to *Cribrihabitans marinus*, this relationship received low bootstrap support, and the two strains shared an AAI value of 75.3%, which falls within the intermediate range for genus delineation (Fig. S2C). Therefore, the distinct phylogenomic placement of *L. marinistellae* from both the *Lutimaribacter* type-species lineage and *Cribrihabitans* provides the primary evidence supporting its reclassification into a novel genus, with the AAI value serving as complementary genomic evidence. These findings indicate that *Lutimaribacter marinistellae* is genomically and phylogenetically distinct from both *Lutimaribacter* and *Cribrihabitans*, supporting its reclassification into a novel genus within the family *Roseobacteraceae*. In contrast, *Cribrihabitans pelagius* KCTC 42981^T^ and *Cribrihabitans neustonicus* JCM 19537^T^ formed a well-supported clade within the genus *Leisingera* (99% bootstrap support; Fig. 2). Consistent with this phylogenomic placement, both species shared high AAI values with *Leisingera methylohalidivorans* DSM 14336^T^, the type species of the genus *Leisingera* (81.8% and 81.5%, respectively; Fig. S2C). Their phylogenomic placement and AAI values therefore justify assignment of *Cribrihabitans pelagius* and *Cribrihabitans neustonicus* to *Leisingera*.

Furthermore, *Primorskyibacter aestuariivivens* OITF-36^T^, which was clearly separated from the remaining members of the genus *Primorskyibacter*, clustered within the genus *Marinovum* with strong bootstrap support (92%; Fig. 2). Consistent with this phylogenomic placement, it shared an AAI value of 76.8% with *Marinovum algicola* FF3^T^, the type species of the genus *Marinovum*, exceeding the genus boundary proposed by Nicholson et al. (2020) (Fig. S2C). Together, these findings support the transfer of *Primorskyibacter aestuariivivens* to the genus *Marinovum*. In contrast, strain S113^T^ shared only 70.8% AAI with *Marinovum algicola* FF3^T^, well below the proposed genus boundary, and formed a distinct phylogenomic lineage separate from *Marinovum*. These findings indicate that strain S113^T^ represents a novel genus rather than a member of *Marinovum*.

Likewise, *Thalassococcus profundi* WRAS1^T^, *Thalassococcus arenae* CAU 1522^T^, and *Thalassococcus lentus* KCTC 32084^T^ each formed independent phylogenomic lineages distinct from both the *Thalassococcus* sensu stricto clade containing the type species and other related genera (Fig. 2). *Thalassococcus profundi* WRAS1^T^ was positioned adjacent to *Puniceibacterium* with 92% bootstrap support but remained distinct from the lineage containing its type species, *Puniceibacterium antarcticum* SM1211^T^. Although the two strains shared an AAI value of 75.6%, which falls within the intermediate range for genus delineation (Fig. S2C), the distinct phylogenomic placement of *Thalassococcus profundi* provides the primary evidence for its reclassification into a novel genus within the family *Roseobacteraceae*, with AAI and other genome-relatedness indices serving as complementary evidence. Similarly, *Thalassococcus arenae* CAU 1522^T^ and *Thalassococcus lentus* KCTC 32084^T^ each formed independent phylogenomic lineages and shared AAI values of ≤ 74.3% and ≤ 75.7%, respectively, with their closest relatives (Fig. S2C). Their distinct phylogenomic placements, together with these genome-relatedness values as complementary evidence, support their recognition as representatives of two additional novel genera within the family *Roseobacteraceae*.

To further evaluate the consistency of the proposed taxonomic framework, pairwise genome-relatedness indices (ANI, AAI, and POCP) were compared among members of the genera affected by the proposed reclassifications before and after taxonomic revision (Fig. 3). Under the current taxonomy, intra- and intergeneric comparisons showed substantial overlap across all three indices, resulting in poorly resolved genus boundaries (Fig. 3A–3C). In contrast, the proposed reclassifications markedly reduced this overlap and produced clearer separation between intra- and intergeneric comparisons. This improvement was consistently observed in the AAI-POCP, AAI-ANI, and ANI-POCP relationships, indicating greater concordance between phylogenomic topology and genome-relatedness indices (Fig. 3D–3F). Collectively, these results provide independent quantitative support for the proposed taxonomic revisions and demonstrate that the revised classification more accurately reflects the evolutionary relationships within the family *Roseobacteraceae*.

### Phenotypic, physiological, and biochemical characteristics

Strain W99^T^ grew well on MA and also on NA, R2A agar, TSA, and LB agar supplemented with approximately 2% (w/v) NaCl. In contrast, strain S113^T^ grew only on MA, whereas strain S268^T^ grew on both MA and R2A agar. Cells of strains W99^T^, S113^T^, and S268^T^ were Gram-stain-negative, non-motile rods measuring approximately 1.0–1.1 × 2.8–2.9 µm, 0.6–0.7 × 1.2–1.3 µm, and 0.7–0.8 × 1.4–1.5 µm, respectively (Fig. S3). None of the strains possessed flagella or exhibited gliding motility, and all were obligately aerobic.

Strains W99^T^, S113^T^, and S268^T^ shared many phenotypic, physiological, and biochemical characteristics with their closely related species, including positive reactions for oxidase and catalase and negative reactions for indole production, glucose fermentation, arginine dihydrolase activity, hydrolysis of casein, Tween 20, and Tween 80, and assimilation of potassium gluconate and capric acid. However, they differed from their closest relatives in cell motility, nitrate reduction, urease and *β*-galactosidase activities, hydrolysis of esculin, gelatin, starch, and tyrosine, and assimilation of several substrates, including ᴅ-glucose, ʟ-arabinose, ᴅ-mannose, ᴅ-mannitol, *N*-acetyl-glucosamine, ᴅ-maltose, adipic acid, malic acid, trisodium citrate, and phenylacetic acid (Table 1).

### Chemotaxonomic characteristics

All three isolates contained Q-10 as the only detectable respiratory quinone, consistent with closely related members of the family *Roseobacteraceae* (Nedashkovskaya et al., 2025; Park et al., 2016; Yi and Chun, 2006). The major fatty acids (> 5.0%) were 11-methyl C_18:1_ *ω*7*c* and summed feature 8 in strain W99^T^; C_16:0_ and summed feature 8 in strain S113^T^; and 11-methyl C_18:1_ *ω*7*c*, cyclo-C_19:0_ *ω*8*c*, and summed feature 8 in strain S268^T^. Detailed cellular fatty acid compositions of the three isolates and their closely related reference strains are presented in Table S4. The polar lipid profile of strain W99^T^ comprised PG, PC, one unidentified phospholipid (PL), and one unidentified aminolipid (AL), consistent with those of *Shimia* species and *Thiosulfatihalobacter marinus* (Wu et al., 2022). The major polar lipids of strain S113^T^ were PG, PC, one AL, and one unidentified lipid (L), whereas strain S268^T^ contained PG, PC, DPG, one PL, and two ALs (Fig. S4).

### Taxonomic conclusion

The combined phylogenetic, phylogenomic, genome-based, phenotypic, biochemical, and chemotaxonomic evidence demonstrates that strain W99^T^ represents a novel species of the genus *Shimia*, for which the name *Shimia rhodophyticola* sp. nov. is proposed. Likewise, strains S113^T^ and S268^T^ represent two novel genus-level lineages within the family *Roseobacteraceae* and are proposed as *Neomarinovum algicola* gen. nov., sp. nov. and *Phycorhabdus gracilariae* gen. nov., sp. nov., respectively. In addition, comprehensive phylogenomic and genome-based relatedness analyses support the transfer of *Thiosulfatihalobacter marinus* to the genus *Shimia*. Because retention of the specific epithet *marinus* would create a later homonym of the pre-existing *Shimia marina* Choi and Cho 2006, the replacement name *Shimia oceanica* nom. nov. is proposed. The analyses also support the transfer of *Cribrihabitans pelagius* and *Cribrihabitans neustonicus* to the genus *Leisingera* as *Leisingera pelagia* comb. nov. and *Leisingera neustonica* comb. nov., respectively, and *Primorskyibacter aestuariivivens* to the genus *Marinovum* as *Marinovum aestuariivivens* comb. nov.

Furthermore, several phylogenetically and genomically distinct lineages are proposed as novel genera within the family *Roseobacteraceae*. These include *Parashimia* gen. nov. for five species currently assigned to *Shimia* (*S. litoralis*, '*S. fortis*', *S. thalassica*, *S. gijangensis*, and *S. sediminis*), *Pararuegeria* gen. nov. for four species currently assigned to *Ruegeria* (*R. discodermiae*, *R. jejuensis*, *R. spongiae*, and *R. hyattellae*), *Alterishimia* gen. nov. for *Shimia biformata*, *Neoshimia* gen. nov. for *Shimia ponticola*, *Paracribrihabitans* gen. nov. for *Lutimaribacter marinistellae*, *Parathalassococcus* gen. nov. for *Thalassococcus arenae*, *Neothalassococcus* gen. nov. for *Thalassococcus lentus*, and *Allothalassococcus* gen. nov. for *Thalassococcus profundi*. A summary of the current and proposed taxonomy of the affected members of the family *Roseobacteraceae* is presented in Table 2.

Following the transfer of *Primorskyibacter aestuariivivens*, an emended description of the genus *Marinovum* is proposed. An emended description of the genus *Shimia* is also provided to reflect its revised circumscription following the transfer of *Thiosulfatihalobacter marinus* and the reassignment of several species to newly established genera.

### Description of *Shimia rhodophyticola* sp. nov.

*Shimia rhodophyticola* (rho.do.phy.ti′co.la. N.L. neut. pl. n. *Rhodophyta*, the division of the red algae; L. suffix. -*cola* (from L. masc. or fem. n. *incola*), inhabitant, dweller; N.L. fem. n. *rhodophyticola*, inhabitant of *Rhodophyta*).

Colonies on MA are cream, circular, and smooth. Cells are Gram-stain-negative, obligately aerobic, and non-flagellated rods without motility. Oxidase- and catalase-positive. Growth occurs at 15–40°C (optimum, 25°C), at pH 7.0–10.0 (optimum, pH 8.0), and in the presence of 0–7.0% (w/v) NaCl (optimum, 2.0%). Nitrate is reduced to nitrite, whereas indole production and glucose fermentation are negative. Hydrolyzes tyrosine, urea, and gelatin, but not casein, Tween 20, Tween 80, esculin, or starch. Arginine dihydrolase and *β*-galactosidase activities are absent. Assimilates ᴅ-glucose, adipic acid, malic acid, trisodium citrate, and phenylacetic acid, but not ʟ-arabinose, ᴅ-mannose, ᴅ-mannitol, *N*-acetyl-glucosamine, ᴅ-maltose, potassium gluconate, or capric acid. Q-10 is the sole respiratory quinone. Major cellular fatty acids (> 5.0%) are 11-methyl C_18:1_ *ω*7*c* and summed feature 8. The polar lipids comprise PG, PC, an unidentified phospholipid, and an unidentified aminolipid.

The type strain is W99^T^ (= KACC 24154^T^ = JCM 38196^T^), isolated from the phycosphere of the marine red alga *Schizymenia* sp., collected from a coastal region in South Korea. The genome size of the strain is 4,262 kb, and its DNA G + C content is 60.5 mol%, as determined from the whole genome sequence. The GenBank accession numbers for the 16S rRNA gene and the genome sequences of strain W99^T^ are PV993570 and JBSQBU000000000, respectively.

### Description of *Neomarinovum* gen. nov.

*Neomarinovum* (Ne.o.ma.rin.o′vum. Gr. masc. adj. *neos*, new; N.L. neut. n. *Marinovum*, a bacterial genus name; N.L. neut. n. *Neomarinovum*, the new *Marinovum*).

Cells are Gram-stain-negative, aerobic, non-motile, and non-flagellated rods. Catalase- and oxidase-positive. Nitrate is not reduced. Q-10 is the major respiratory quinone. Major fatty acids (> 5.0%) are C_16:0_ and summed feature 8. Major polar lipids comprise PG and PC. Phylogenetically, the genus is a member of the family *Roseobacteraceae* within the order *Rhodobacterales* of the phylum *Pseudomonadota*. The type species is *Neomarinovum algicola*.

### Description of *Neomarinovum algicola* sp. nov.

*Neomarinovum algicola* (al.gi′co.la. L. fem. n. *alga*, an alga; L. masc./fem. n. suff. -*cola*, inhabitant, dweller; from L. masc./fem. n. *incola*, dweller; N.L. masc./fem. n. *algicola*, an alga dweller).

In addition to the characteristics given for the genus, colonies on MA are grayish-pink, circular, and smooth. Cells are short rods (0.6–0.7 µm wide and 1.2–1.3 µm long) and do not exhibit gliding motility. Growth occurs at 20–30°C (optimum, 25°C), at pH 7.0–9.0 (optimum, pH 8.0), and in the presence of 1.0–3.0% (w/v) NaCl (optimum, 2.0%). Indole production and glucose fermentation are negative. Hydrolyzes tyrosine, but not gelatin, casein, Tween 20, Tween 80, esculin, or starch. Positive for *β*-galactosidase activity, but negative for urease and arginine dihydrolase activities. Does not assimilate ᴅ-glucose, ʟ-arabinose, ᴅ-mannose, ᴅ-mannitol, *N*-acetyl-glucosamine, ᴅ-maltose, potassium gluconate, capric acid, adipic acid, malic acid, trisodium citrate, or phenylacetic acid. Additional polar lipids include one unidentified aminolipid and one unidentified lipid.

The type strain is S113^T^ (= KACC 23417^T^ = JCM 38210^T^), isolated from the phycosphere of the marine green alga *Chaetomorpha* sp., collected from a coastal region in South Korea. The genome size of the strain is 4,314 kb, and its DNA G + C content is 60.0 mol%, as determined from the whole genome sequence. The GenBank accession numbers for the 16S rRNA gene and the genome sequences of strain S113^T^ are OR234759 and JBSQBV000000000, respectively.

### Description of *Phycorhabdus* gen. nov.

*Phycorhabdus* (Phy.co.rhab′dus. Gr. neut. n. *phykos*, algae; Gr. fem. n. *rhabdos*, a rod; N.L. fem. n. *Phycorhabdus*, a rod-shaped organism associated with algae).

Cells are Gram-stain-negative, aerobic, non-motile, and non-flagellated rods. Catalase- and oxidase-positive. Nitrate is reduced to nitrite. Q-10 is the major respiratory quinone. Major cellular fatty acids (> 5.0%) are 11-methyl C_18:1_ *ω*7*c*, cyclo-C_19:0_ *ω*8*c*, and summed feature 8. Major polar lipids comprise PG, PC, and DPG. Phylogenetically, the genus is a member of the family *Roseobacteraceae* within the order *Rhodobacterales* of the phylum *Pseudomonadota*. The type species is *Phycorhabdus gracilariae*.

### Description of *Phycorhabdus gracilariae* sp. nov.

*Phycorhabdus gracilariae* (gra.ci.la′ri.ae. N.L. gen. fem. n. *gracilariae*, of the red alga *Gracilaria*).

In addition to the characteristics given for the genus, colonies on MA are pink, circular, and smooth. Cells are short rods (0.7–0.8 µm wide and 1.4–1.5 µm long) and do not exhibit gliding motility. Growth occurs at 15–40°C (optimum, 30°C), at pH 7.0–11.0 (optimum, pH 8.0), and in the presence of 1.0–7.0% (w/v) NaCl (optimum, 2.0%). Indole production and glucose fermentation are negative. Does not hydrolyze esculin, gelatin, casein, Tween 20, Tween 80, starch, or tyrosine. Positive for urease and *β*-galactosidase activities, but negative for arginine dihydrolase activity. Weakly assimilates ᴅ-maltose and malic acid, but does not assimilate ᴅ-glucose, ʟ-arabinose, ᴅ-mannose, ᴅ-mannitol, *N*-acetyl-glucosamine, potassium gluconate, capric acid, adipic acid, trisodium citrate, or phenylacetic acid. Additional polar lipids include one unidentified phospholipid and two unidentified aminolipids.

The type strain is S268^T^ (= KACC 24156^T^ = JCM 38211^T^), isolated from the phycosphere of the marine red alga *Gracilaria* sp., collected from a coastal region in South Korea. The genome size of the strain is 3,864 kb, and its DNA G + C content is 56.1 mol%, as determined from the whole genome sequence. The GenBank accession numbers for the 16S rRNA gene and the genome sequences of strain S268^T^ are PV993569 and JBWOEC000000000, respectively.

### Description of *Pararuegeria* gen. nov.

*Pararuegeria* (Pa.ra.rue.ge′ri.a. Gr. prep. *para*, beside, alongside of, near; N.L. fem. n. *Ruegeria*, a bacterial generic name; N.L. fem. n. *Pararuegeria*, a genus alongside, or closely related to, the genus *Ruegeria*).

Cells are Gram-stain-negative, aerobic, and ovoid to short rod-shaped. Motility by flagella is variable. Oxidase and catalase are positive, whereas nitrate reduction is variable. The genomic DNA G + C content ranges from 59.0 to 60.8 mol%. The predominant respiratory quinone is Q-10. The major cellular fatty acids (> 5.0%) are C_12:0_ 3-OH, C_16:0_ 2-OH, and summed feature 8. The major polar lipids are PC and PG, whereas PE and DPG are variably present among species. Phylogenetically, the genus belongs to the family *Roseobacteraceae* within the order *Rhodobacterales* of the phylum *Pseudomonadota*. The type species is *Pararuegeria spongiae*.

### Description of *Pararuegeria discodermiae* comb. nov.

*Pararuegeria discodermiae* (dis.co.der′mi.ae. N.L. gen. n. *discodermiae*, of the sponge genus *Discodermia*).

Basonym: *Ruegeria discodermiae* Moon et al. 2026 (Oren and Göker, 2026).

The description of *Pararuegeria discodermiae* is identical to that proposed for *Ruegeria discodermiae* (Moon et al., 2025). The type strain is 2205SS24-7^T^ (= KACC 23237^T^ = LMG 33064^T^).

### Description of *Pararuegeria jejuensis* comb. nov.

*Pararuegeria jejuensis* (je.ju.en′sis. N.L. fem. adj. *jejuensis*, Jeju island, Republic of Korea, from where the type strain was isolated).

Basonym: *Ruegeria jejuensis* Moon et al. 2026 (Oren and Göker, 2026).

The description of *Pararuegeria jejuensis* is identical to that proposed for *Ruegeria jejuensis* (Moon et al., 2025). The type strain is 2304DJ69-11^T^ (= KACC 23805^T^ = MCCC 1K09534^T^).

### Description of *Pararuegeria spongiae* comb. nov.

*Pararuegeria spongiae* (spon′gi.ae. L. gen. n. *spongiae*, of a sponge, source of the type strain).

Basonym: *Ruegeria spongiae* Han and Park 2023.

The description of *Pararuegeria spongiae* is identical to that proposed for *Ruegeria spongiae* (Han and Park, 2023). The type strain is 2012CJ41-6^T^ (= KACC 22645^T^ = LMG 32585^T^).

### Description of *Pararuegeria hyattellae* comb. nov.

*Pararuegeria hyattellae* (hy.at.tel′lae. N.L. gen. n. *hyattellae*, of the sponge genus *Hyattella*).

Basonym: *Ruegeria hyattellae* Moon et al. 2026 (Oren and Göker, 2026).

The description of *Pararuegeria hyattellae* is identical to that proposed for *Ruegeria hyattellae* (Moon et al., 2025). The type strain is 2012CJ15-1^T^ (= KACC 23842^T^ = MCCC 1K09526^T^).

### Description of *Paracribrihabitans* gen. nov.

*Paracribrihabitans* (Pa.ra.cri.bri.ha′bi.tans. Gr. prep. *para*, beside, alongside of, near; N.L. masc. n. *Cribrihabitans*, a bacterial generic name; N.L. masc. n. *Paracribrihabitans*, a genus alongside, or closely related to, the genus *Cribrihabitans*).

Cells are Gram-stain-negative, aerobic, and motile rods with flagella. Catalase- and oxidase-positive. Nitrate is not reduced. Q-10 is the major respiratory quinone. Major fatty acids (> 5.0%) are 11-methyl C_18:1_ *ω*7*c* and summed feature 8. Major polar lipids comprise PG, DPG, and PE. Phylogenetically, the genus is a member of the family *Roseobacteraceae* within the order *Rhodobacterales* of the phylum *Pseudomonadota*. The type species is *Paracribrihabitans marinistellae*.

### Description of *Paracribrihabitans marinistellae* comb. nov.

*Paracribrihabitans marinistellae* (ma.ri.ni.stel′lae. L. masc. adj. *marinus*, from the sea; L. fem. n. *stella*, star; N.L. gen. n. *marinistellae*, of a sea star).

Basonym: *Lutimaribacter marinistellae* Zhang et al. 2016.

The description of *Paracribrihabitans marinistellae* is identical to that proposed for *Lutimaribacter marinistellae* (Zhang et al., 2016). The type strain is SF-12^T^ (= KCTC 42911^T^ = MCCC 1K01154^T^).

### Description of *Leisingera pelagia* comb. nov.

*Leisingera pelagia* (pe.la′gi.a. L. fem. adj. *pelagia*, belonging to the sea).

Basonym: *Cribrihabitans pelagius* Yoon et al. 2016.

The description of *Leisingera pelagia* is identical to that proposed for *Cribrihabitans pelagius* (Yoon et al., 2016). The type strain is KMU-32^T^ (= KCTC 42981^T^ = NBRC 111834^T^).

### Description of *Leisingera neustonica* comb. nov.

*Leisingera neustonica* (neus.to′ni.ca. N.L. fem. adj. *neustonica*, pertaining to and living in the neuston).

Basonym: *Cribrihabitans neustonicus* Hameed et al. 2015.

The description of *Leisingera neustonica* is identical to that proposed for *Cribrihabitans neustonicus* (Hameed et al., 2014). The type strain is CC-AMHB-3^T^ (= BCRC 80695^T^ = JCM 19537^T^).

### Description of *Alterishimia* gen. nov.

*Alterishimia* (Al.te.ri.shi′mi.a. L. masc. adj. *alter*, another, other; N.L. fem. n. *Shimia*, a bacterial genus name; N.L. fem. n. *Alterishimia*, another *Shimia*).

Cells are Gram-stain-negative, strictly aerobic, and non-flagellated rods. Catalase- and oxidase-positive. Nitrate is reduced to nitrite. Q-10 is the major respiratory quinone. The major cellular fatty acids (> 5.0%) are C_16:0_, 11-methyl C_18:1_ *ω*7*c*, and summed feature 8. The major polar lipids comprise DPG, PG, and PC. Phylogenetically, the genus is a member of the family *Roseobacteraceae* within the order *Rhodobacterales* of the phylum *Pseudomonadota*. The type species is *Alterishimia biformata*.

### Description of *Alterishimia biformata* comb. nov.

*Alterishimia biformata* (bi.for.ma′ta. L. fem. adj. *biformata*, double-formed, two-shaped, pertaining to the different cell morphology during different growth phases).

Basonym: *Shimia biformata* Hameed et al. 2013.

The description of *Alterishimia biformata* is identical to that proposed for *Shimia biformata* (Hameed et al., 2013). The type strain is CC-AMW-C^T^ (= BCRC 80548^T^ = JCM 18818^T^).

### Description of *Parashimia* gen. nov.

*Parashimia* (Pa.ra.shi′mi.a. Gr. prep. *para*, beside, alongside of, near; N.L. fem. n. *Shimia*, a bacterial generic name; N.L. fem. n. *Parashimia*, a genus alongside, or closely related to, the genus *Shimia*).

Cells are Gram-stain-negative, aerobic, and rod- or ovoid-shaped. Motility is variable. Catalase activity and nitrate reduction are variable. The genomic DNA G + C content ranges from 47.0 to 59.8 mol%. The major respiratory quinone is Q-10. The major cellular fatty acids (> 5.0%) commonly include summed feature 8. The major polar lipids commonly comprise PG, whereas PC, PE, DPG, and phosphatidyl-*N*,*N*-dimethylethanolamine are variably present depending on the strain. Phylogenetically, the genus is a member of the family *Roseobacteraceae* within the order *Rhodobacterales* of the phylum *Pseudomonadota*. The type species is *Parashimia litoralis*.

### Description of *Parashimia litoralis* comb. nov.

*Parashimia litoralis* (li.to.ra′lis. L. fem. adj. *litoralis*, of the shore).

Basonym: *Shimia litoralis* (Kim et al. 2008) Zhang et al. 2023.

The description of *Parashimia litoralis* is identical to that proposed for *Pelagicola litoralis* (Kim et al., 2008). The type strain is CL-ES2^T^ (= DSM 18290^T^ = KCCM 42274^T^).

### Description of *Parashimia fortis* sp. nov.

*Parashimia fortis* (for′tis. L. fem. adj. *fortis*, strong, referring to the fact that the strain is still viable after six months of laboratory cultivation).

Colonies on MA are beige, circular, convex, and smooth. Cells are Gram-stain-negative, aerobic, non-motile, and ovoid. Growth occurs at 20–37°C (optimum, 28–35°C) and pH 6.0–9.0 (optimum, pH 7.0) and in the presence of 1.0–7.0% (w/v) NaCl (optimum, 3.0–4.0%). Catalase activity is negative. Positive for alkaline phosphatase, esterase (C4), esterase lipase (C8), leucine arylamidase, and naphthol-AS-BI-phosphohydrolase activities, and weakly positive for valine arylamidase activity. Hydrolyzes starch, alginate, and casein, but does not hydrolyze CM-cellulose or Tweens 20, 40, 60, or 80. Acids are produced from ᴅ-ribose, fructose, ʟ-sorbose, esculin, ᴅ-tagatose, and potassium 5-ketogluconate, and weakly from ᴅ-arabinose, ʟ-arabinose, ᴅ-xylose, ʟ-xylose, ᴅ-turanose, ᴅ-lyxose, and potassium 2-ketogluconate. Utilizes glucuronamide, ʟ-malic acid, bromo-succinic acid, acetic acid, acetoacetic acid, and α-hydroxybutyric acid as sole carbon sources, and weakly utilizes ᴅ-glucuronic acid. Q-10 is the sole respiratory quinone. Major cellular fatty acids (> 5.0%) are C_16:0_, C_17:0_, 11-methyl C_18:1_ *ω*7*c*, and summed feature 8 (C_18:1_ *ω*7*c* and/or C_18:1_ *ω*6*c*). The polar lipids comprise PG, PME, DPG, and PE.

The type strain is SDUM112013^T^ (= KCTC 8307^T^ = MCCC 1H01481^T^), which was isolated from intertidal sediments of Xiaoshi Island, China. The genome size of the strain is 3,823 kb, and its DNA G + C content is 59.8 mol%, as determined from the whole genome sequence. The GenBank accession numbers for the 16S rRNA gene and the genome sequences of strain SDUM112013^T^ are PP892595 and JBBPEU000000000, respectively.

### Description of *Parashimia thalassica* comb. nov.

*Parashimia thalassica* (tha.las′si.ca. Gr. fem. n. *thalassa*, the sea; L. fem. adj. suff. -*ica*, suffix used with the sense of pertaining to; L. fem. adj. *thalassica*, pertaining to the sea, of the sea).

Basonym: *Shimia thalassica* Arahal et al. 2019.

The description of *Parashimia thalassica* is identical to that proposed for *Shimia thalassica* (Arahal et al., 2019). The type strain is R47^T^ (= CECT 7735^T^ = LMG 29909^T^).

### Description of *Parashimia gijangensis* comb. nov.

*Parashimia gijangensis* (gi.jang.en′sis. N.L. fem. adj. *gijangensis*, pertaining to Gijang, the location of the National Fisheries Research and Development Institute, where the type strain was characterized).

Basonym: *Shimia gijangensis* (Kim et al. 2014) Arahal et al. 2019.

The description of *Parashimia gijangensis* is identical to that proposed for *Pseudopelagicola gijangensis* (Kim et al., 2014). The type strain is YSS-7^T^ (= KCTC 42049^T^ = CECT 8540^T^).

### Description of *Parashimia sediminis* comb. nov.

*Parashimia sediminis* (se.di′mi.nis. L. gen. n. *sediminis*, of sediment).

Basonym: *Shimia sediminis* Zhu et al. 2021.

The description of *Parashimia sediminis* is identical to that proposed for *Shimia sediminis* (Zhu et al., 2021). The type strain is ZQ172^T^ (= KCTC 62578^T^ = MCCC 1K03531^T^).

### Description of *Shimia oceanica* nom. nov.

*Shimia oceanica* (o.ce.a′ni.ca. N.L. fem. adj. *oceanica*, of or pertaining to the ocean).

Basonym: *Thiosulfatihalobacter marinus* Wu et al. 2022.

The description of *Shimia oceanica* is identical to that proposed for *Thiosulfatihalobacter marinus* (Wu et al., 2022). The type strain is GL-11-2^T^ (= KCTC 82723^T^ = MCCC M20691^T^).

### Emended description of the genus *Shimia* Choi and Cho 2006

Emended description of the genus *Shimia* is as given previously by Choi and Cho (2006), with the following amendments. The major fatty acid is typically summed feature 8. Depending on the species, C_16:0_, 11-methyl C_18:1_ *ω*7*c*, or cyclo-C_19:0_ *ω*8*c* may occur as additional major fatty acids.

### Description of *Neoshimia* gen. nov.

*Neoshimia* (Ne.o.shi′mi.a. Gr. masc. adj. *neos*, new; N.L. fem. n. *Shimia*, a bacterial genus name; N.L. fem. n. *Neoshimia*, the new *Shimia*).

Cells are Gram-stain-negative, aerobic, non-motile, and non-flagellated rods. Catalase- and oxidase-negative. Nitrate is not reduced. Q-10 is the major respiratory quinone. Major fatty acids (> 5.0%) are C_18:0_, 11-methyl C_18:1_ *ω*7*c*, C_10:0_ 3-OH, and summed feature 8. Major polar lipids comprise PC, PE, and PG. Phylogenetically, the genus is a member of the family *Roseobacteraceae* within the order *Rhodobacterales* of the phylum *Pseudomonadota*. The type species is *Neoshimia ponticola*.

### Description of *Neoshimia ponticola* comb. nov.

*Neoshimia ponticola* (pon.ti′co.la. L. masc. n. *pontus*, the sea; L. suff. -*cola* (from L. masc. or fem. n. *incola*) inhabitant, dweller; N.L. fem. n. *ponticola*, a dweller of sea).

Basonym: *Shimia ponticola* Chen et al. 2023.

The description of *Neoshimia ponticola* is identical to that proposed for *Shimia ponticola* (Chen et al., 2023). The type strain is WX04^T^ (= KCTC 62628^T^ = MCCC 1K02295^T^).

### Description of *Marinovum aestuariivivens* comb. nov.

*Marinovum aestuariivivens* (aes.tu.a.ri.i.vi′vens. L. neut. n. *aestuarium* (gen. *aestuarii*), tidal flat; L. pres. part. *vivens*, living; N.L. masc. part. adj. *aestuariivivens*, living in a tidal flat).

Basonym: *Primorskyibacter aestuariivivens* Park et al. 2016.

The description of *Marinovum aestuariivivens* is identical to that proposed for *Primorskyibacter aestuariivivens* (Park et al., 2016). The type strain is OITF-36^T^ (= KCTC 52455^T^ = NBRC 112419^T^).

### Emended description of the genus *Marinovum* Martens et al. 2006S

The description of the genus is as given previously by Lafay et al. (1995) and Martens et al. (2006), with the following amendments. The major fatty acid is C_18:1_ *ω*7*c*. Major polar lipids are typically PG, PE, and PC; depending on the species, additional polar lipids such as DPG or phosphatidylmonomethylethanolamine may also be present.

### Description of *Allothalassococcus* gen. nov.

*Allothalassococcus* (Al.lo.tha.las.so.coc′cus. Gr. adj. *allos*, other; N.L. masc. n. *Thalassococcus*, a bacterial genus name; N.L. masc. n. *Allothalassococcus*, another *Thalassococcus*).

Cells are Gram-stain-negative, aerobic, non-motile, and rod- or ovoid-shaped. Catalase- and oxidase-positive. Nitrate is not reduced. Q-10 is the major respiratory quinone. Major fatty acids (> 5.0%) are C_12:1_ 3-OH, C_16:0_, and summed feature 8. Major polar lipids are PC, PG, and PE. Phylogenetically, the genus is a member of the family *Roseobacteraceae* within the order *Rhodobacterales* of the phylum *Pseudomonadota*. The type species is *Allothalassococcus profundi*.

### Description of *Allothalassococcus profundi* comb. nov.

*Allothalassococcus profundi* (pro.fun′di. L. gen. n. *profundi*, of/ from the depths of the sea).

Basonym: *Thalassococcus profundi* Yu et al. 2019.

The description of *Allothalassococcus profundi* is identical to that proposed for *Thalassococcus profundi* (Yu et al., 2019). The type strain is WRAS1^T^ (= KCTC 52696^T^ = MCCC 1K03253^T^).

### Description of *Parathalassococcus* gen. nov.

*Parathalassococcus* (Pa.ra.tha.las.so.coc′cus. Gr. prep. *para*, beside; N.L. masc. n. *Thalassococcus*, a bacterial genus name; N.L. masc. n. *Parathalassococcus*, a genus beside *Thalassococcus*).

Cells are Gram-stain-negative, aerobic, and non-motile rods. Catalase- and oxidase-positive. Nitrate is reduced. Q-10 is the major respiratory quinone. Major fatty acids (> 5.0%) are 11-methyl C_18:1_ *ω*7*c*, C_18:0_, C_17:0_, and summed feature 8. Major polar lipids are PC, PE, and PG. Phylogenetically, the genus is a member of the family *Roseobacteraceae* within the order *Rhodobacterales* of the phylum *Pseudomonadota*. The type species is *Parathalassococcus arenae*.

### Description of *Parathalassococcus arenae* comb. nov.

*Parathalassococcus arenae* (a.re′nae. L. gen. n. *arenae*, of sand).

Basonym: *Thalassococcus arenae* Weerawongwiwat et al. 2024 (Oren and Göker, 2024).

The description of *Parathalassococcus arenae* is identical to that proposed for *Thalassococcus arenae* (Weerawongwiwat et al., 2022). The type strain is CAU 1522^T^ (= KCTC 72545^T^ = MCCC 1K04064^T^).

### Description of *Neothalassococcus* gen. nov.

*Neothalassococcus* (Ne.o.tha.las.so.coc′cus. Gr. masc. adj. *neos*, new; N.L. masc. n. *Thalassococcus*, a bacterial genus name; N.L. masc. n. *Neothalassococcus*, a new *Thalassococcus*).

Cells are Gram-stain-negative, aerobic, non-motile, and rod- or ovoid-shaped. Catalase-positive and oxidase-negative. Nitrate is not reduced. Q-10 is the major respiratory quinone. Major fatty acids (> 5.0%) are 11-methyl C_18:1_ *ω*7*c*, C_18:1_ *ω*7*c*, and C_18:0_. Major polar lipids are PC and PG. Phylogenetically, the genus is a member of the family *Roseobacteraceae* within the order *Rhodobacterales* of the phylum *Pseudomonadota*. The type species is *Neothalassococcus lentus*.

### Description of *Neothalassococcus lentus* comb. nov.

*Neothalassococcus lentus* (len′tus. L. masc. adj. *lentus*, slow, delayed, referring to the slow growth of the microorganism).

Basonym: *Thalassococcus lentus* Park et al. 2013.

The description of *Neothalassococcus lentus* is identical to that proposed for *Thalassococcus lentus* (Park et al., 2013). The type strain is YCS-24^T^ (= CCUG 62791^T^ = KCTC 32084^T^).

## Figures and Tables

**Fig. 1.**
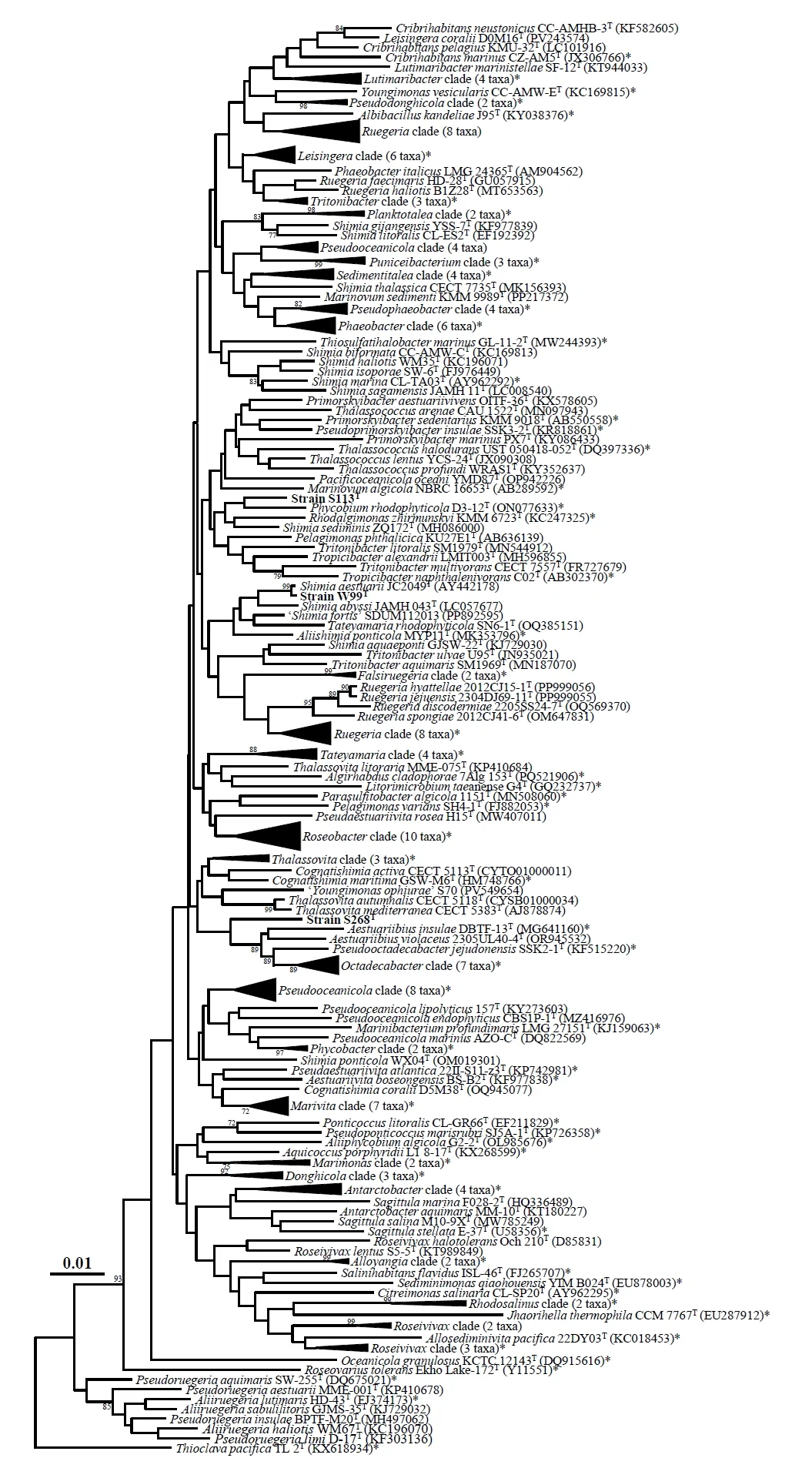
Neighbor-joining tree based on 16S rRNA gene sequences showing the phylogenetic positions of strains W99^T^, S113^T^, and S268^T^ (shown in bold) within the family *Roseobacteraceae*. Bootstrap values > 70%, calculated from 1,000 replicates, are shown at the nodes. *Thioclava pacifica* TL 2^T^ (KX618934) was used as the outgroup. Type species are indicated by asterisks, and black triangles represent collapsed clades containing the corresponding type species. The scale bar indicates 0.01 nucleotide substitutions per site.

**Fig. 2.**
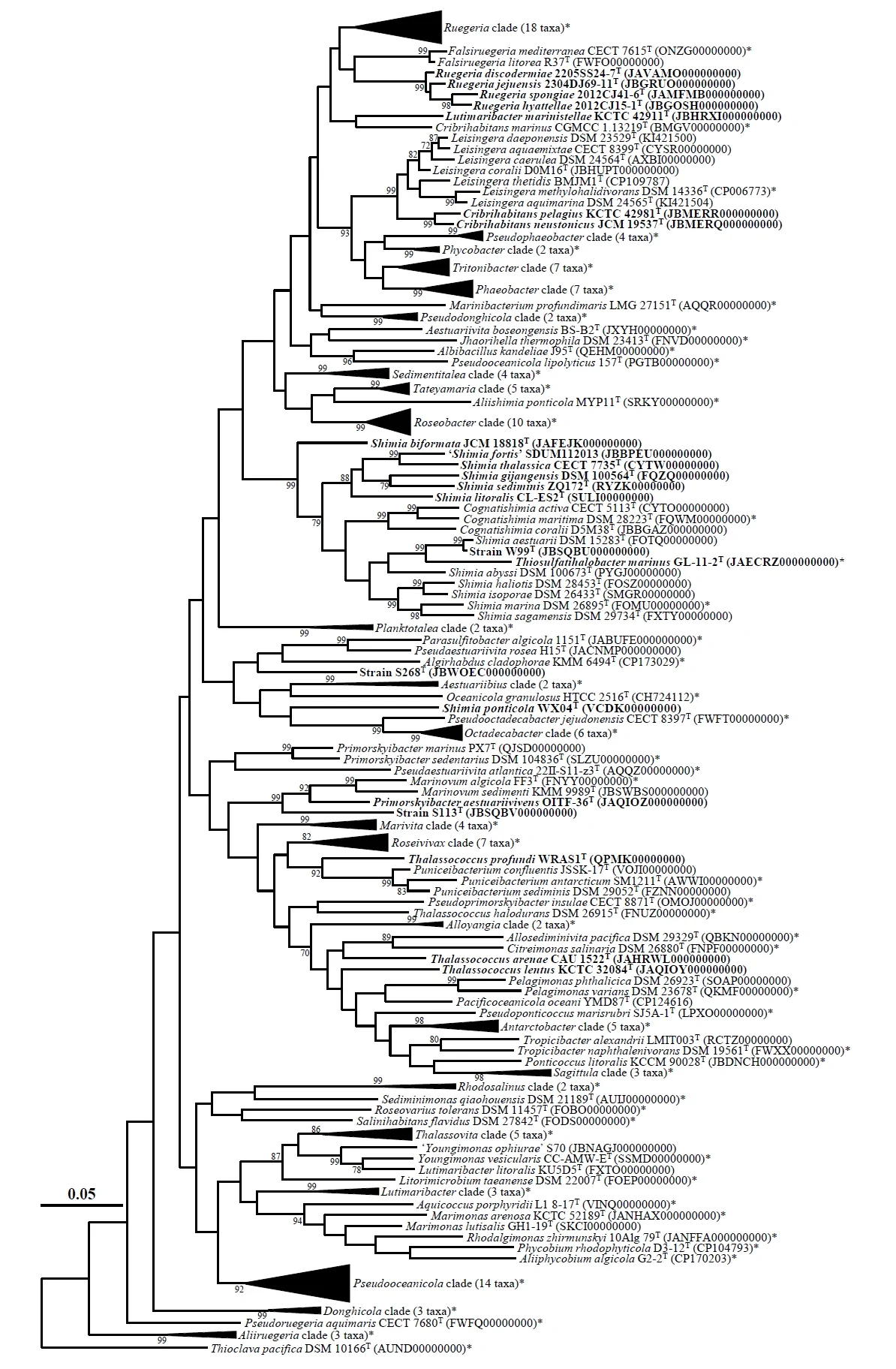
Maximum-likelihood phylogenomic tree showing the positions of strains W99^T^, S113^T^, and S268^T^ (shown in bold) within the family *Roseobacteraceae*, based on the concatenated amino acid sequences of 120 bacterial marker genes identified by GTDB-Tk. Bootstrap values > 70%, calculated from 1,000 replicates, are shown at the nodes. *Thioclava pacifica* DSM 10166^T^ (AUND00000000) was used as the outgroup. Type species are indicated by asterisks, and black triangles represent collapsed clades containing the corresponding type species. Taxa that may warrant taxonomic reclassification based on the phylogenomic topology are also shown in bold. The scale bar indicates 0.05 amino acid substitutions per site.

**Fig. 3.**
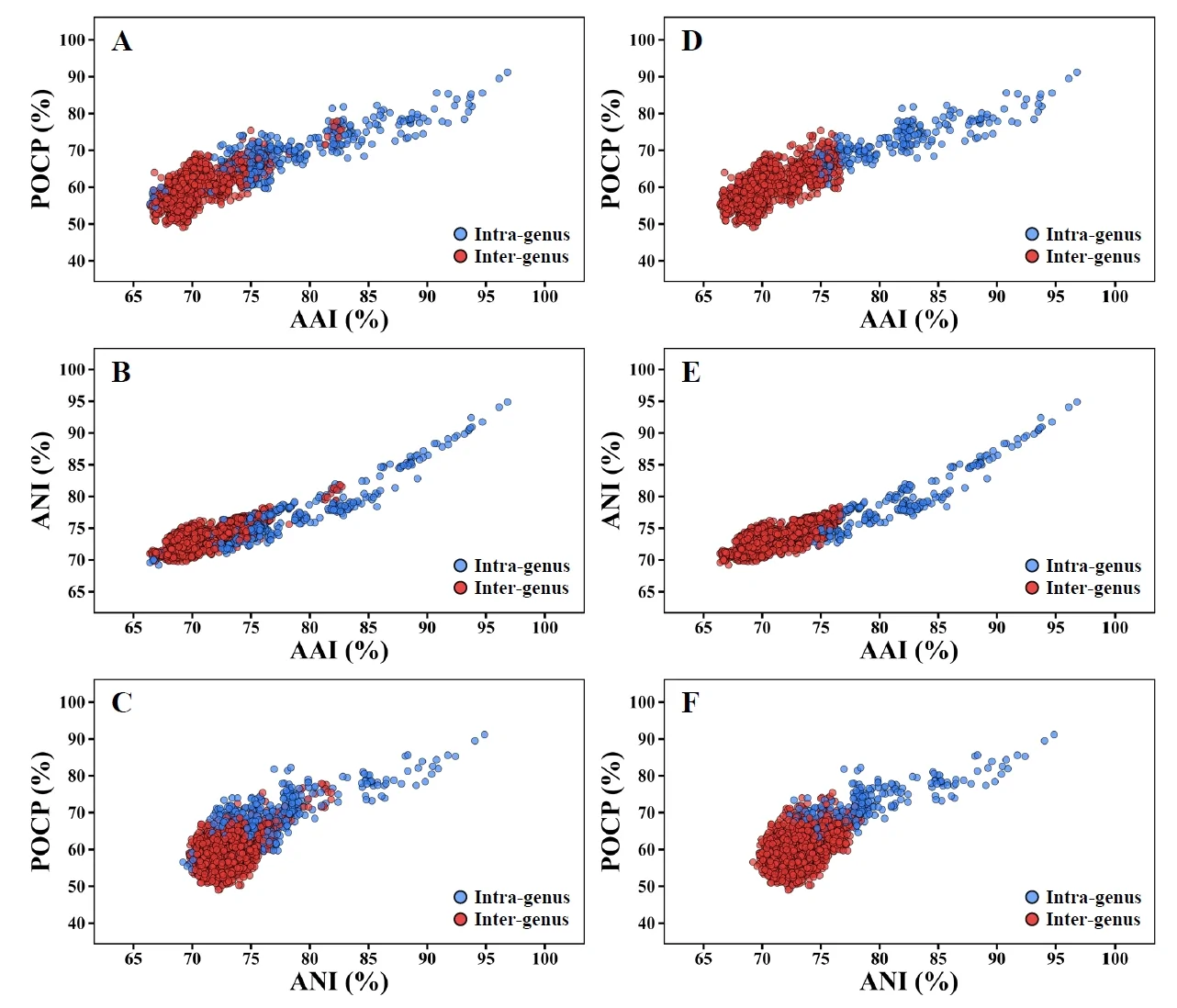
Pairwise comparisons of genome-relatedness indices among members of genera affected by the proposed taxonomic revisions within the family *Roseobacteraceae*, based on the current and proposed classifications. Relationships between average amino acid identity (AAI) and percentage of conserved proteins (POCP) (A, D), AAI and average nucleotide identity (ANI) (B, E), and ANI and POCP (C, F) are shown for the current taxonomy (A–C) and the proposed taxonomy (D–F). Red and blue symbols represent intergeneric and intrageneric comparisons, respectively.

**Table 1.** Differential phenotypic characteristics of strains W99^T^, S113^T^, and S268^T^ and closely related taxa within the family *Roseobacteraceae* Taxa: 1, strain W99ᵀ (this study); 2, strain S113ᵀ (this study); 3, strain S268ᵀ (this study); 4, *Shimia aestuarii* KCTC 12049^T^ (Wirth and Whitman, 2018; Yi and Chun, 2006); 5, *Thiosulfatihalobacter marinus* KCTC 82723^T^ (Wu et al., 2022); 6, *Marinovum algicola* JCM 21266^T^ (Lafay et al., 1995; Martens et al., 2006); 7, *Primorskyibacter aestuariivivens* KCTC 52455^T^ (Park et al., 2016). All strains are positive for oxidase and catalase activities^*^. All strains are negative for the following characteristics: indole production^*^, glucose fermentation^*^, hydrolysis^*^ of casein, Tween 20, and Tween 80, arginine dihydrolase activity^*^, and assimilation^*^ of potassium gluconate and capric acid. +, positive; –, negative; *w*, weakly positive; ND, no data.

Characteristic	1	2	3	4	5	6	7
Isolation source	Marine alga	Marine alga	Marine alga	Tidal sediment	Seawater	Dinoflagellate	Tidal flat
Colony color	Cream	Grayish pink	Pink	Cream	Cream	White	Greyish yellow
Cell morphology	Short rod	Short rod	Short rod	Ovoid	Rod	Ovoid rod	Ovoid or rod
Cell motility	−	−	−	−	−	+	+
Growth condition:							
Temperature (°C, optimum)	15–40 (25)	20–30 (25)	15–40 (30)	15–35 (35)	4–40 (30)	10–37 (25–30)	10–40 (30)
pH (optimum)	7.0–10.0 (8.0)	7.0–9.0 (8.0)	7.0–11.0 (8.0)	6.0–11.0 (7.0)	6.0–8.0 (6.5–7.0)	6.0–9.0 (7.5)	6.0–8.0 (7.0–8.0)
NaCl (%, optimum)	0–7.0 (2.0)	1.0–3.0 (2.0)	1.0–7.0 (2.0)	1.0–7.0 (2.0)	0.5–7.0 (2.0)	0.6–11.7 (ND)	0–9.0 (2.0–3.0)
Reduction of nitrate	+	−	+	+	−	−	+
Hydrolysis* of:							
Esculin	−	−	−	−	−	+	+
Gelatin	+	−	−	−	−	−	−
Starch	−	−	−	−	−	+	+
Tyrosine	+	+	−	+	*w*	+	+
Activity* of:							
Urease	+	−	+	−	−	−	−
*β*-Galactosidase	−	+	+	−	−	+	−
Assimilation* of:							
ᴅ-Glucose	+	−	−	*w*	−	+	−
ʟ-Arabinose	−	−	−	−	−	+	−
ᴅ-Mannose	−	−	−	−	−	+	−
ᴅ-Mannitol	−	−	−	−	−	*+*	−
*N*-Acetyl-glucosamine	−	−	−	−	*w*	*+*	−
ᴅ-Maltose	−	−	*w*	−	−	*w*	−
Adipic acid	*w*	−	−	+	+	*w*	*w*
Malic acid	+	−	*w*	+	+	+	+
Trisodium citrate	+	−	−	+	*w*	+	*w*
Phenylacetic acid	+	−	−	+	*w*	*w*	−
Major polar lipids^†^	PC, PG	PC, PG	PC, PG, DPG	ND	PC, PG, PE, DPG	ND	PC, PG, PE, PME

* These analyses were conducted under the same conditions in this study. ^†^PC, phosphatidylcholine; PG, phosphatidylglycerol; DPG, diphosphatidylglycerol; PE, phosphatidylethanolamine; PME, phosphatidylmonomethylethanolamine.

**Table 2.** Original, revised, and proposed taxonomy of species within the family *Roseobacteraceae*. The proposed taxonomic changes are based on the phylogenomic and genome-based relatedness analyses performed in this study. –, no taxonomic change.

Original taxonomy	Revised taxonomy	Proposed taxonomy
*Ruegeria discodermiae* Moon et al. 2026	–	*Pararuegeria discodermiae* comb. nov.
*Ruegeria jejuensis* Moon et al. 2026	–	*Pararuegeria jejuensis* comb. nov.
*Ruegeria spongiae* Han and Park 2023	–	*Pararuegeria spongiae* gen. nov., comb. nov.
*Ruegeria hyattellae* Moon et al. 2026	–	*Pararuegeria hyattellae* comb. nov.
*Lutimaribacter marinistellae* Zhang et al. 2016	–	*Paracribrihabitans marinistellae* gen. nov., comb. nov.
*Cribrihabitans pelagius* Yoon et al. 2016	–	*Leisingera pelagia* comb. nov.
*Cribrihabitans neustonicus* Hameed et al. 2014	–	*Leisingera neustonica* comb. nov.
*Shimia biformata* Hameed et al. 2013	–	*Alterishimia biformata* gen. nov., comb. nov.
'*Shimia fortis*' Dong et al. 2026	–	*Parashimia fortis* sp. nov.
*Shimia thalassica* Arahal et al. 2019	–	*Parashimia thalassica* comb. nov.
*Pseudopelagicola gijangensis* Kim et al. 2014	*Shimia gijangensis* Arahal et al. 2019	*Parashimia gijangensis* comb. nov.
*Shimia sediminis* Zhu et al. 2021	–	*Parashimia sediminis* comb. nov.
*Pelagicola litoralis* Kim et al. 2008	*Shimia litoralis* Zhang et al. 2023	*Parashimia litoralis* gen. nov., comb. nov.
*Thiosulfatihalobacter marinus* Wu et al. 2022	–	*Shimia oceanica* nom. nov.
*Shimia ponticola* Chen et al. 2023	–	*Neoshimia ponticola* gen. nov., comb. nov.
*Primorskyibacter aestuariivivens* Park et al. 2016	–	*Marinovum aestuariivivens* comb. nov.
*Thalassococcus profundi* Yu et al. 2019	–	*Allothalassococcus profundi* gen. nov., comb. nov.
*Thalassococcus arenae* Weerawongwiwat et al. 2024	–	*Parathalassococcus arenae* gen. nov., comb. nov.
*Thalassococcus lentus* Park et al. 2013	–	*Neothalassococcus lentus* gen. nov., comb. nov.

## Supplementary Information

Table S1. Potential ecological distributions of 16S rRNA gene sequences closely related to strains W99^T^, S113^T^, and S268^T^, assessed using the Integrated Microbial Next-Generation Sequencing (IMNGS) platform at a 99.0% sequence similarity threshold. “Matched no. (Prevalence)” represents the number (percentage) of datasets containing 16S rRNA gene sequences showing ≥ 99.0% sequence similarity to the respective query sequences of strain W99^T^, S113^T^, and S268^T^, and “ARA” denotes the average relative abundance of the matched sequences within the datasets. Prevalence values below 0.5% for all three query sequences are not shown. –, not detected.

Table S2. General genomic features of strains W99^T^, S113^T^, and S268^T^ and closely related type strains within the family *Roseobacteraceae*

Table S3. Average nucleotide identity (ANI; lower-left triangle) and digital DNA–DNA hybridization (dDDH; upper-right triangle) values among strains W99^T^, S113^T^, and S268^T^ and their closely related type strains

Table S4. Cellular fatty acid compositions (%) of strains W99^T^, S113^T^, and S268^T^ and closely related type strains

Fig. S1. Maximum-likelihood (ML; A) and maximum-parsimony (MP; B) trees based on 16S rRNA gene sequences showing the phylogenetic positions of strains W99^T^, S113^T^, and S268^T^ (shown in bold) within the family *Roseobacteraceae*. Bootstrap values > 70%, calculated from 1,000 replicates, are shown at the nodes. *Thioclava pacifica* TL 2^T^ (KX618934) was used as the outgroup. Type species are indicated by asterisks, and black triangles represent collapsed clades containing the corresponding type species. Scale bars represent nucleotide substitutions per site in the ML tree and the number of character changes across the full 16S rRNA gene sequence in the MP tree.

Fig. S2. Average amino acid identity (AAI) values among strains W99^T^ and S268^T^ and their closely related taxa (A), among strain S113^T^ and its closely related taxa (B), and among taxa of other clades in the phylogenomic tree (C). Dendrograms were generated by hierarchical clustering of AAI values using the ComplexHeatmap package (version 2.14.0) in R.

Fig. S3. Transmission electron micrograph showing the general morphology of negatively stained cells of strains W99^T^ (A), S113^T^ (B), and S268^T^ (C). Cells were grown on marine agar at their respective optimum temperatures for 3 days and negatively stained with UranyLess.

Fig. S4. Two-dimensional thin-layer chromatograms (TLC) showing the polar lipid profile of strains W99^T^, S113^T^, and S268^T^. Solvent systems: (I) chloroform-methanol-water (65:25:4, v/v/v) and (II) chloroform-acetic acid-methanol-water (80:15:12:4, v/v/v/v). The TLC plates were sprayed with 10% ethanolic molybdophosphoric acid (A), ninhydrin (B), Dittmer-Lester (C), and *α*-naphthol/sulfuric acid (D) reagents for the detection of total polar lipids, aminolipids, phospholipids, and glycolipids, respectively. PG, phosphatidylglycerol; PC, phosphatidylcholine; DPG, diphosphatidylglycerol; PL, unidentified phospholipid; AL, unidentified aminolipid; L, unidentified lipid.
