# Supplementary material for: Shimia rhodophyticola sp. nov., Neomarinovum algicola gen. nov., sp. nov., and Phycorhabdus gracilariae gen. nov., sp. nov., isolated from marine algae, and the taxonomic revision of nineteen taxa within the family Roseobacteraceae
Source: J Microbiol. 2026 Oct 31;64(10):e2607010. doi: 10.71150/jm.2607010 (PMC13720383; doi:10.71150/jm.2607010)
Supplement: Supplementary file 1 [file jm-2607010-Supplementary-Fig-S1.pdf]

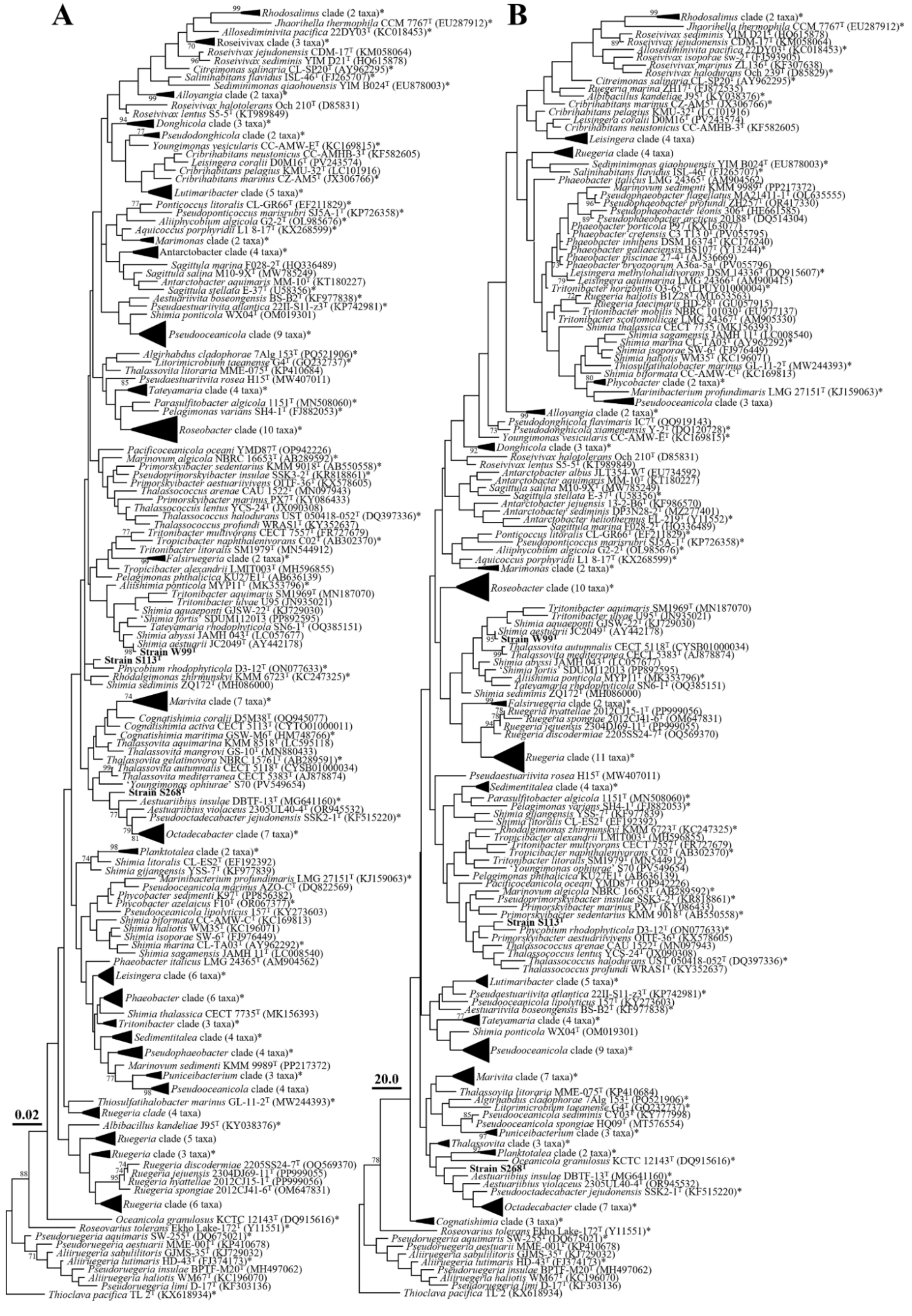

Fig. S1. Maximum-likelihood (ML; A) and maximum-parsimony (MP; B) trees based on 16S rRNA gene sequences showing the phylogenetic positions of strains W99^{T}, S113^{T}, and S268^{T} (shown in bold) within the family *Roseobacteraceae*. Bootstrap values > 70%, calculated from 1,000 replicates, are shown at the nodes. *Thioclava pacifica* TL 2^{T} (KX618934) was used as the outgroup. Type species are indicated by asterisks, and black triangles represent collapsed clades containing the corresponding type species. Scale bars represent nucleotide substitutions per site in the ML tree and the number of character changes across the full 16S rRNA gene sequence in the MP tree.
