# Supplementary material for: Shimia rhodophyticola sp. nov., Neomarinovum algicola gen. nov., sp. nov., and Phycorhabdus gracilariae gen. nov., sp. nov., isolated from marine algae, and the taxonomic revision of nineteen taxa within the family Roseobacteraceae
Source: J Microbiol. 2026 Oct 31;64(10):e2607010. doi: 10.71150/jm.2607010 (PMC13720383; doi:10.71150/jm.2607010)
Supplement: Supplementary file 2 [file jm-2607010-Supplementary-Fig-S2.pdf]

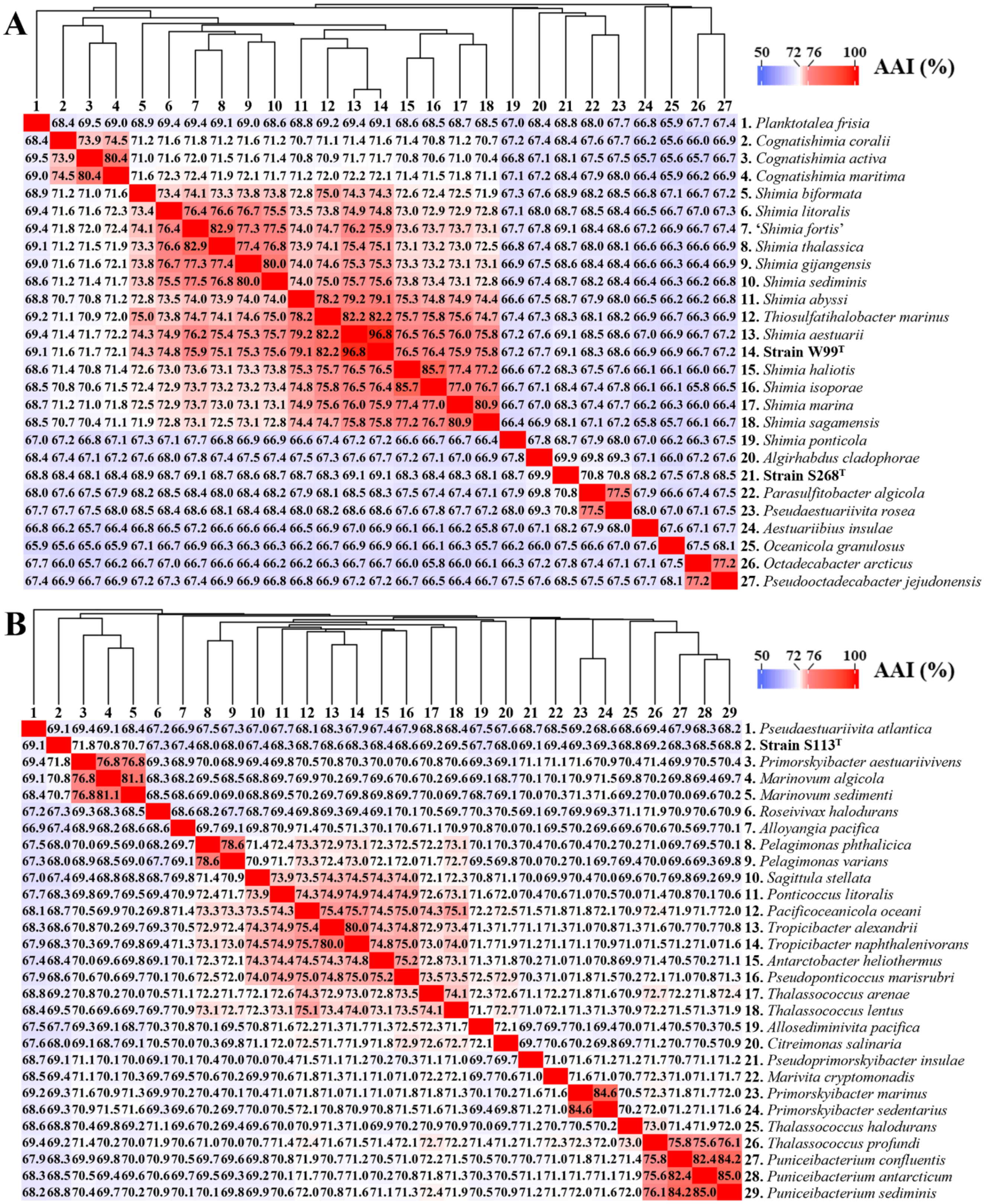

Fig. S2. Average amino acid identity (AAI) values among strains W99^{T} and S268^{T} and their closely related taxa (A), among strain S113^{T} and its closely related taxa (B), and among taxa of other clades in the phylogenomic tree (C). Dendrograms were generated by hierarchical clustering of AAI values using the ComplexHeatmap package (version 2.14.0) in R.

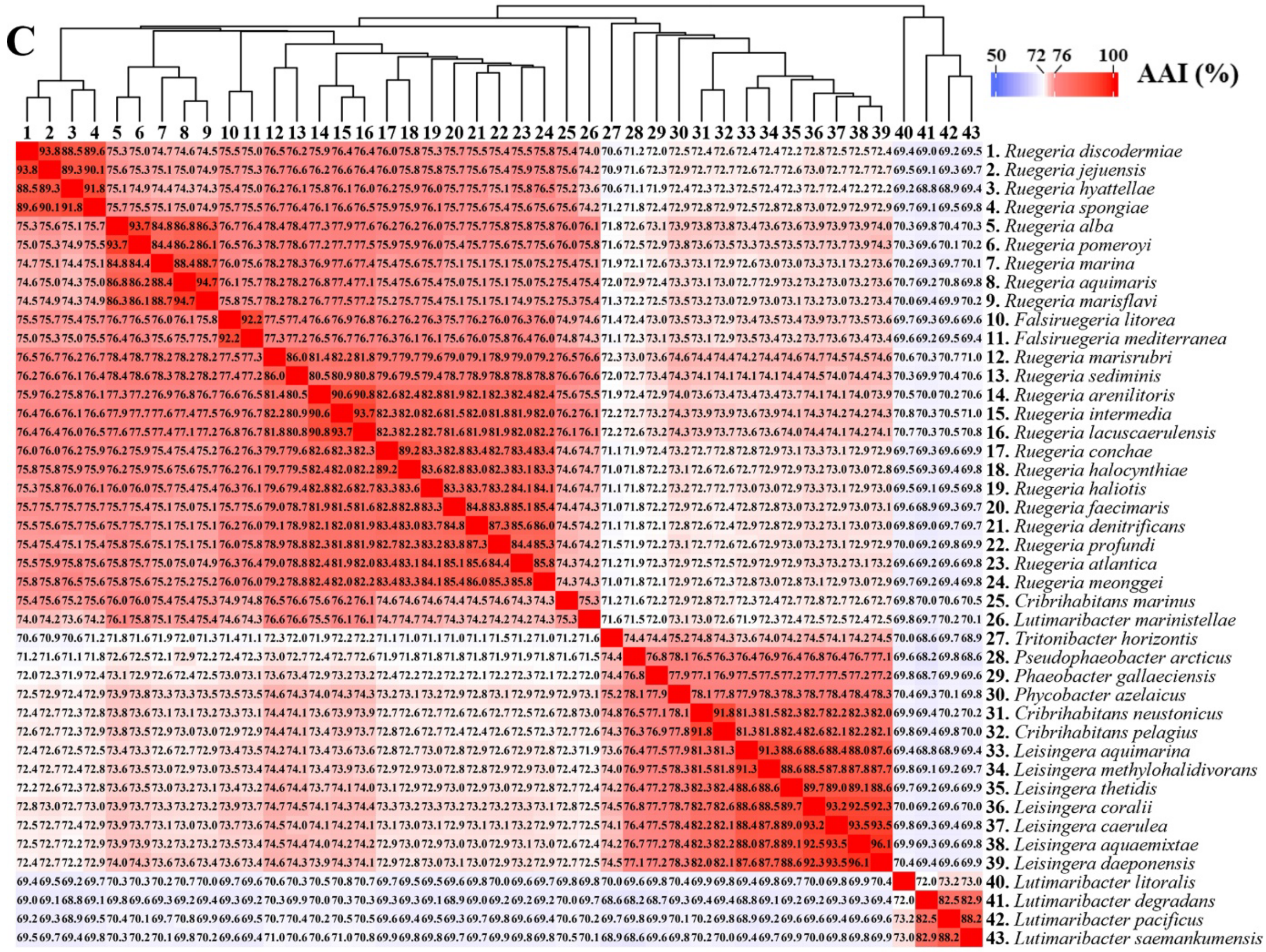

Fig. 2. (Continued)
