# Supplementary material for: Shimia rhodophyticola sp. nov., Neomarinovum algicola gen. nov., sp. nov., and Phycorhabdus gracilariae gen. nov., sp. nov., isolated from marine algae, and the taxonomic revision of nineteen taxa within the family Roseobacteraceae
Source: J Microbiol. 2026 Oct 31;64(10):e2607010. doi: 10.71150/jm.2607010 (PMC13720383; doi:10.71150/jm.2607010)
Supplement: Supplementary file 3 [file jm-2607010-Supplementary-Fig-S3.pdf]

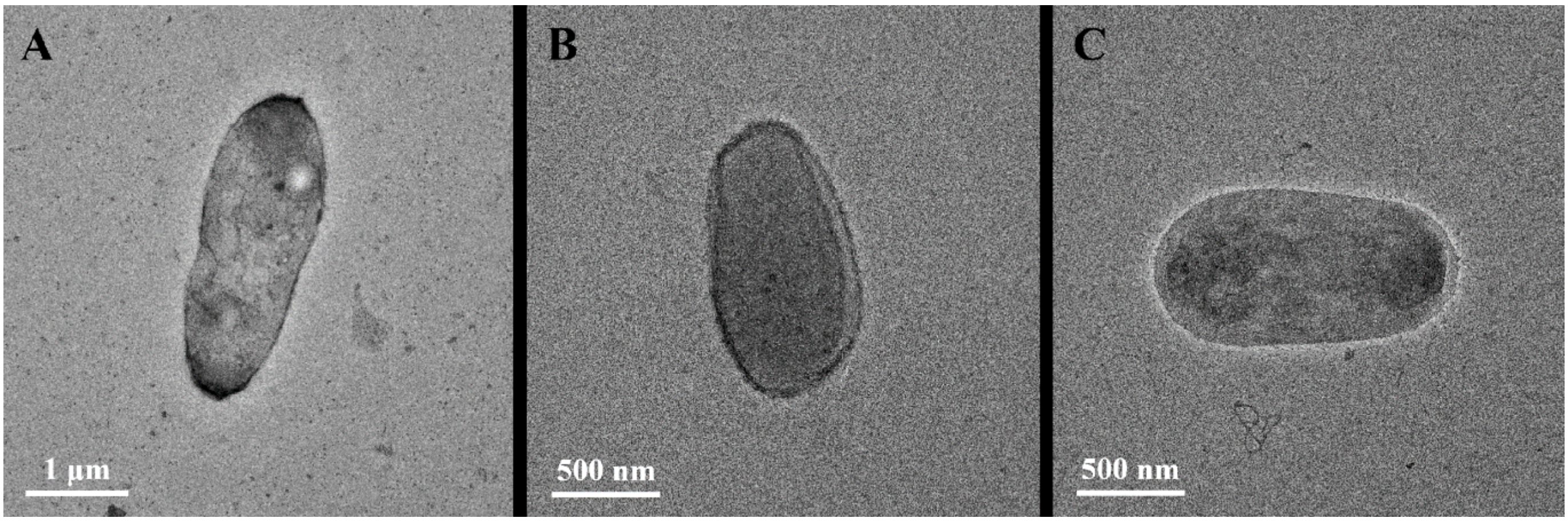

Fig. S3. Transmission electron micrograph showing the general morphology of negatively stained cells of strains W99^T (A), S113^T (B), and S268^T (C). Cells were grown on marine agar at their respective optimum temperatures for 3 days and negatively stained with UranyLess.
