# Supplementary material for: Shimia rhodophyticola sp. nov., Neomarinovum algicola gen. nov., sp. nov., and Phycorhabdus gracilariae gen. nov., sp. nov., isolated from marine algae, and the taxonomic revision of nineteen taxa within the family Roseobacteraceae
Source: J Microbiol. 2026 Oct 31;64(10):e2607010. doi: 10.71150/jm.2607010 (PMC13720383; doi:10.71150/jm.2607010)
Supplement: Supplementary file 4 [file jm-2607010-Supplementary-Fig-S4.pdf]

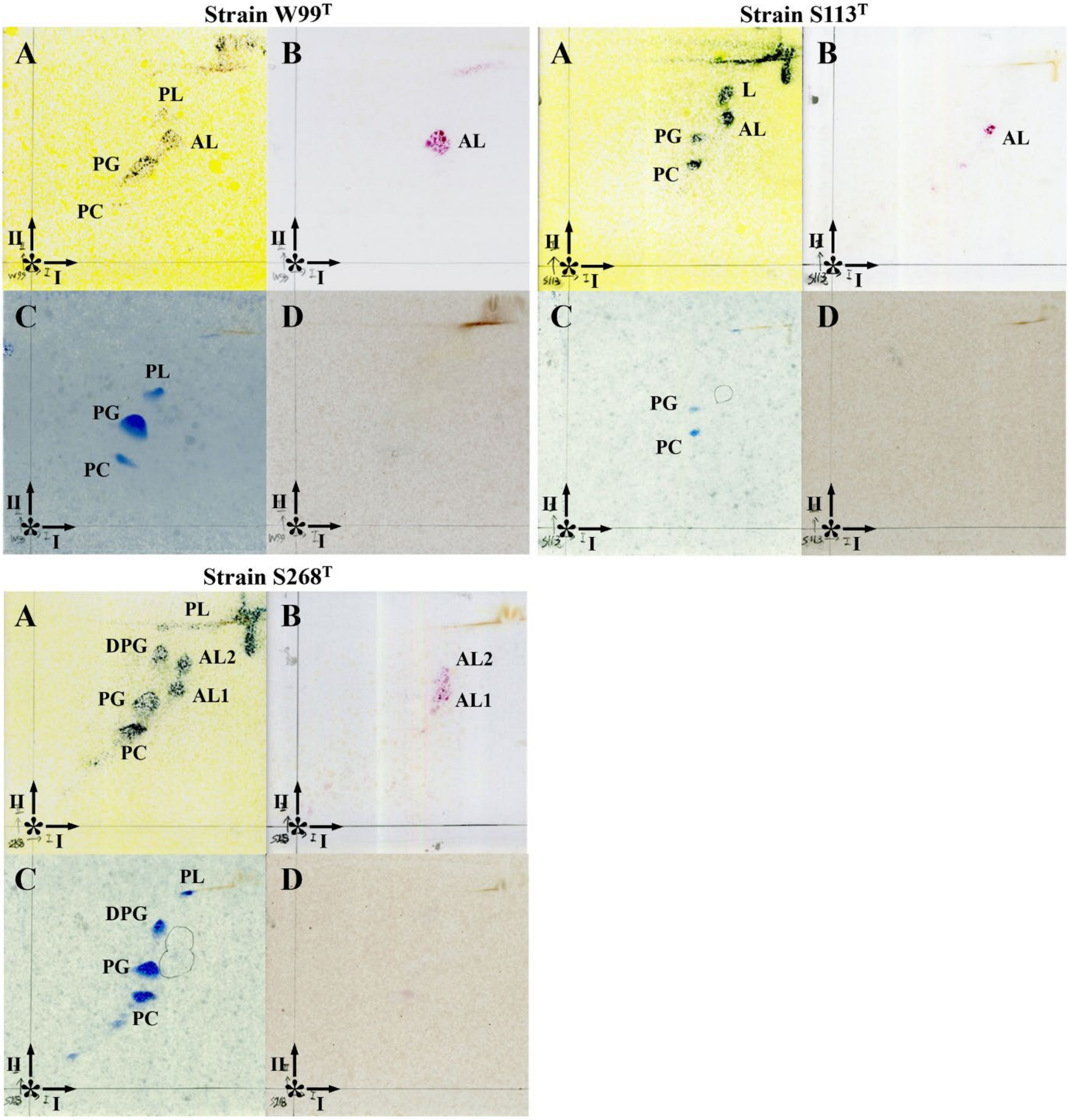

Fig. S4. Two-dimensional thin-layer chromatograms (TLC) showing the polar lipid profile of strains W99^T, S113^T, and S268^T. Solvent systems: (I) chloroform-methanol-water (65:25:4, v/v/v) and (II) chloroform-acetic acid-methanol-water (80:15:12:4, v/v/v/v). The TLC plates were sprayed with 10% ethanolic molybdophosphoric acid (A), ninhydrin (B), Dittmer-Lester (C), and α-naphthol/sulfuric acid (D) reagents for the detection of total polar lipids, aminolipids, phospholipids, and glycolipids, respectively. PG, phosphatidylglycerol; PC, phosphatidylcholine; DPG, diphosphatidylglycerol; PL, unidentified phospholipid; AL, unidentified aminolipid; L, unidentified lipid.
