# Supplementary material for: Shimia rhodophyticola sp. nov., Neomarinovum algicola gen. nov., sp. nov., and Phycorhabdus gracilariae gen. nov., sp. nov., isolated from marine algae, and the taxonomic revision of nineteen taxa within the family Roseobacteraceae
Source: J Microbiol. 2026 Oct 31;64(10):e2607010. doi: 10.71150/jm.2607010 (PMC13720383; doi:10.71150/jm.2607010)
Supplement: Supplementary file 5 [file jm-2607010-Supplementary-Table-S1.pdf]

Table S1. Potential ecological distributions of 16S rRNA gene sequences closely related to strains W99^T, S113^T, and S268^T, assessed using the Integrated Microbial Next-Generation Sequencing (IMNGS) platform at a 99.0% sequence similarity threshold. "Matched no. (Prevalence)" represents the number (percentage) of datasets containing 16S rRNA gene sequences showing ≥ 99.0% sequence similarity to the respective query sequences of strain W99^T, S113^T, and S268^T, and "ARA" denotes the average relative abundance of the matched sequences within the datasets. Prevalence values below 0.5% for all three query sequences are not shown. –, not detected.

**Source/host**	**No. of datasets**	**Strain W99^T**		**Strain S113^T**		**Strain S268^T**	
		**Matched no. (prevalence, %)**	**ARA (%)**	**Matched no. (prevalence, %)**	**ARA (%)**	**Matched no. (prevalence, %)**	**ARA (%)**
Capasa incensata	1	–	–	1 (100)	0.003	–	–
Sea anemone metagenome	43	33 (76.7)	3.082	33 (76.7)	3.082	–	–
Tripneustes gratilla	26	14 (53.8)	0.045	18 (69.2)	0.166	6 (23.1)	0.060
Seminavis robusta	9	4 (44.4)	0.251	8 (88.9)	0.574	1 (11.1)	0.033
Leptogorgia sarmentosa	3	–	–	1 (33.3)	0.008	–	–
Cancer borealis	31	7 (22.6)	0.001	3 (9.7)	< 0.001	1 (3.2)	< 0.001
Panulirus ornatus	20	3 (15.0)	0.346	4 (20.0)	0.501	–	–
Fungia	41	–	–	2 (4.9)	0.192	1 (2.4)	0.190
Rumen bacterium 1/9293-11A	12	1 (8.3)	0.001	–	–	–	–
Calliphora terraenovae	31	1 (3.2)	0.001	–	–	–	–
Coral metagenome	2,795	85 (3.0)	0.075	106 (3.8)	0.078	49 (1.8)	0.090
Urban metagenome	35	1 (2.9)	< 0.001	–	–	–	–
Annelid metagenome	37	1 (2.7)	< 0.001	1 (2.7)	< 0.001	–	–
Unidentified virus	77	2 (2.6)	< 0.001	–	–	–	–
Human blood metagenome	84	2 (2.4)	0.011	–	–	–	–
Shrimp gut metagenome	86	2 (2.3)	0.001	–	–	–	–

Beach sand metagenome	180	3 (1.7)	< 0.001	1 (0.6)	< 0.001	–	–
Euprymna scolopes	62	1 (1.6)	< 0.001	–	–	–	–
Epibiont metagenome	132	–	–	2 (1.5)	< 0.001	2 (1.5)	< 0.001
Mollusc metagenome	157	2 (1.3)	0.009	–	–	–	–
Compost metagenome	383	4 (1.0)	0.004	–	–	–	–
Sediment metagenome	4,605	46 (1.0)	< 0.001	7 (0.2)	< 0.001	17 (0.4)	< 0.001
Coral reef metagenome	161	–	–	1 (0.6)	< 0.001	–	–
Marine metagenome	37,438	129 (0.3)	0.006	217 (0.6)	0.029	91 (0.2)	0.003
Crassostrea gigas	1,170	–	–	6 (0.5)	< 0.001	1 (0.1)	< 0.001
Biofilm metagenome	2,953	6 (0.2)	< 0.001	4 (0.1)	< 0.001	15 (0.5)	0.003
Plant metagenome	12,101	4 (0.03)	< 0.001	1 (0.008)	< 0.001	69 (0.6)	< 0.001
Rock metagenome	169	–	–	–	–	1 (0.6)	0.005
