# Supplementary material for: Shimia rhodophyticola sp. nov., Neomarinovum algicola gen. nov., sp. nov., and Phycorhabdus gracilariae gen. nov., sp. nov., isolated from marine algae, and the taxonomic revision of nineteen taxa within the family Roseobacteraceae
Source: J Microbiol. 2026 Oct 31;64(10):e2607010. doi: 10.71150/jm.2607010 (PMC13720383; doi:10.71150/jm.2607010)
Supplement: Supplementary file 6 [file jm-2607010-Supplementary-Table-S2.pdf]

Table S2. General genomic features of strains W99^T, S113^T, and S268^T and closely related type strains within the family *Roseobacteraceae*

Taxa: 1, strain W99^T (JBSQBU000000000); 2, strain S113^T (JBSQBV000000000); 3, strain S268^T (JBWOEC000000000); 4, *Shimia aestuarii* DSM 15283^T (FOTQ00000000); 5, *Thiosulfatihalobacter marinus* GL-11-2^T (JAECRZ000000000); 6, *Marinovum algicola* FF3^T (FNYY00000000); 7, *Primorskyibacter aestuariivivens* OITF-36^T (JAQIOZ000000000).

**Features***	**1**	**2**	**3**	**4**	**5**	**6**	**7**
No. of contigs	4	6	2	27	68	63	44
Genome size (kb)	4,262	4,314	3,864	4,227	4,159	5,397	4,293
G + C content (mol%)	60.5	60.0	56.1	60.4	62.3	66.1	61.5
No. of total genes	4,095	4,198	4,096	4,143	4,098	5,270	4,266
No. of protein-coding sequences	4,009	3,990	3,475	4,046	4,014	5,131	4,171
No. of total RNA genes	56	47	49	62	50	54	53
No. of tRNA genes	47	40	43	50	44	45	46
No. of rRNA (16S, 23S, 5S) operons	2	1	1	3	1	2	1
No. of noncoding RNA genes	3	4	3	3	3	3	4

*Genomic features were obtained from GenBank annotations generated using the NCBI Prokaryotic Genome Annotation Pipeline (PGAP; software revision 6.11). Further information on PGAP is available at https://www.ncbi.nlm.nih.gov/genome/annotation_prok/
