# Supplementary material for: Shimia rhodophyticola sp. nov., Neomarinovum algicola gen. nov., sp. nov., and Phycorhabdus gracilariae gen. nov., sp. nov., isolated from marine algae, and the taxonomic revision of nineteen taxa within the family Roseobacteraceae
Source: J Microbiol. 2026 Oct 31;64(10):e2607010. doi: 10.71150/jm.2607010 (PMC13720383; doi:10.71150/jm.2607010)
Supplement: Supplementary file 7 [file jm-2607010-Supplementary-Table-S3.pdf]

Table S3. Average nucleotide identity (ANI; lower-left triangle) and digital DNA–DNA hybridization (dDDH; upper-right triangle) values among strains W99^T, S113^T, and S268^T and their closely related type strains

Taxa: 1, strain W99^T (JBSQBU000000000); 2, strain S113^T (JBSQBV000000000); 3, strain S268^T (JBWOEC000000000); 4, *Shimia aestuarii* DSM 15283^T (FOTQ00000000); 5, *Thiosulfatihalobacter marinus* GL-11-2^T (JAECRZ000000000); 6, *Shimia abyssi* DSM 100673^T (PYGJ00000000); 7, *Shimia haliotis* DSM 28453^T (FOSZ00000000); 8, *Shimia isoporae* DSM 26433^T (SMGR00000000); 9, *Shimia marina* DSM 26895^T (FOMU00000000); 10, *Shimia sagamensis* DSM 29734^T (FXTY00000000); 11, *Marinovum algicola* FF3^T (FNYY00000000); 12, *Marinovum sedimenti* KMM 9989^T (JBSWBS000000000); 13, *Primorskyibacter aestuariivivens* OITF-36^T (JAQIOZ000000000); 14, *Parasulfitobacter algicola* 1151^T (JABUFE000000000); 15, *Pseudaestuariivita rosea* H15^T (JACNMP000000000); 16, *Aestuariibius insulae* DBTF-13^T (JBFONW000000000).

		**dDDH value (%)**															
		**1**	**2**	**3**	**4**	**5**	**6**	**7**	**8**	**9**	**10**	**11**	**12**	**13**	**14**	**15**	**16**
	**1**	–	21.0	19.6	59.6	21.8	19.4	19.3	19.3	19.6	18.8	21.2	20.2	20.2	19.8	19.3	20.1
	**2**	72.1	–	18.9	20.1	24.9	19.1	19.5	19.2	21.7	19.3	20.3	19.5	20.5	19.0	19.9	18.1
	**3**	70.7	70.3	–	19.8	18.8	19.0	19.5	18.8	19.1	18.6	20.7	20.7	19.3	18.5	19.1	18.9
	**4**	94.9	72.0	70.7	–	21.6	19.4	19.3	19.1	19.6	18.9	20.2	20.1	21.0	19.7	19.8	20.1
	**5**	79.4	72.7	70.5	79.2	–	19.3	19.0	18.8	19.8	18.3	21.0	19.1	20.7	19.5	19.0	19.5
	**6**	75.7	70.4	69.9	75.7	75.6	–	18.4	18.1	19.6	18.7	18.1	19.4	19.2	18.6	19.4	19.8
	**7**	74.5	70.9	70.1	74.7	74.4	73.6	–	20.9	20.8	19.9	19.7	19.5	18.9	18.9	19.2	19.4
**ANI value**	**8**	73.5	70.4	69.8	73.6	73.3	72.3	78.4	–	19.5	19.1	19.3	19.9	19.8	19.0	20.0	19.8
**(%)**	**9**	74.4	71.6	70.0	74.4	74.4	73.5	75.1	74.0	–	19.9	23.1	19.4	21.7	18.1	19.5	19.9
	**10**	73.8	70.5	69.6	73.7	73.4	72.8	75.3	73.9	76.6	–	18.2	18.8	19.3	21.0	19.0	19.5
	**11**	73.8	73.1	71.0	73.7	74.5	71.6	71.9	71.2	73.0	71.4	–	22.2	21.5	19.9	19.2	18.3
	**12**	73.0	72.7	70.7	73.2	73.1	71.8	71.8	70.9	71.6	71.6	79.6	–	20.3	20.6	19.7	18.6
	**13**	73.7	73.2	71.0	73.9	74.5	71.8	72.2	71.1	72.6	71.6	77.3	76.5	–	19.5	19.1	18.7
	**14**	70.2	69.5	70.4	70.2	70.4	69.8	69.6	68.9	69.7	69.2	70.3	70.4	70.5	–	23.4	18.2
	**15**	71.6	70.6	71.0	71.5	71.6	70.4	70.4	69.9	70.5	69.8	71.9	71.4	72.1	76.2	–	19.8
	**16**	70.1	69.8	69.6	69.7	69.8	69.6	70.3	69.1	69.8	69.9	70.1	70.0	70.1	70.4	70.8	–
