# Supplementary material for: Shimia rhodophyticola sp. nov., Neomarinovum algicola gen. nov., sp. nov., and Phycorhabdus gracilariae gen. nov., sp. nov., isolated from marine algae, and the taxonomic revision of nineteen taxa within the family Roseobacteraceae
Source: J Microbiol. 2026 Oct 31;64(10):e2607010. doi: 10.71150/jm.2607010 (PMC13720383; doi:10.71150/jm.2607010)
Supplement: Supplementary file 8 [file jm-2607010-Supplementary-Table-S4.pdf]

Table S4. Cellular fatty acid compositions (%) of strains W99^T, S113^T, and S268^T and closely related type strains

Taxa: 1, strain W99^T; 2, strain S113^T; 3, strain S268^T; 4, *Shimia aestuarii* DSM 15283^T; 5, *Thiosulfatihalobacter marinus* GL-11-2^T; 6, *Marinovum algicola* FF3^T; 7, *Primorskyibacter aestuariivivens* OITF-36^T. All data were obtained in this study. Values are expressed as percentages of the total fatty acids. Fatty acids accounting for less than 1.0% in all strains are omitted. Major fatty acids (> 5.0%) are shown in bold. Symbols: tr, trace (< 1.0%); –, not detected.

Fatty acid	1	2	3	4	5	6	7
Saturated:							
$C_{10:0}$	–	–	–	–	2.2	tr	tr
$C_{12:0}$	tr	1.9	1.1	**11.4**	**24.0**	**18.1**	**27.2**
$C_{16:0}$	4.7	**5.7**	3.1	3.1	**5.5**	2.1	2.3
$C_{17:0}$	1.0	1.8	1.5	tr	–	–	tr
$C_{18:0}$	1.7	3.2	4.3	1.2	1.9	1.9	1.1
Unsaturated:							
$C_{14:1}$ $\omega 5c$	1.4	1.6	2.2	–	–	–	–
iso-$C_{17:1}$ $\omega 10c$	–	–	–	–	–	–	1.2
11-methyl $C_{18:1}$ $\omega 7c$	**8.5**	–	**6.6**	4.0	5.6	4.6	–
cyclo-$C_{19:0}$ $\omega 8c$	–	–	**25.2**	–	8.4	–	–
$C_{20:1}$ $\omega 7c$	tr	–	1.3	tr	tr	1.0	–
Branched:							
iso-$C_{10:0}$	2.8	4.7	–	–	–	–	tr
iso-$C_{19:0}$	–	1.3	–	–	–	–	tr
Hydroxy:							
$C_{8:0}$ 3-OH	tr	1.2	–	–	–	–	–
$C_{10:0}$ 2-OH	–	–	1.5	–	–	–	–
$C_{10:0}$ 3-OH	2.2	–	–	1.9	–	tr	tr
$C_{11:0}$ 2-OH	–	–	1.1	–	–	–	–
$C_{12:0}$ 3-OH	tr	–	–	–	4.1	tr	–
iso-$C_{12:0}$ 3-OH	tr	2.6	–	tr	–	–	tr
$C_{16:0}$ 2-OH	2.7	–	–	3.5	–	–	–
iso-$C_{17:0}$ 3-OH	–	3.1	1.9	–	–	–	tr
Summed feature*:							
3 ($C_{16:1}$ $\omega 7c$ and/or $C_{16:1}$ $\omega 6c$)	tr	–	tr	tr	tr	tr	tr
8 ($C_{18:1}$ $\omega 7c$ and/or $C_{18:1}$ $\omega 6c$)	**68.2**	**56.4**	**44.1**	**72.1**	**41.1**	**69.2**	**62.9**

*Summed features represent groups of two or more fatty acids that cannot be reliably resolved under the chromatographic conditions used and are therefore reported by the MIDI system as a single feature with one percentage of the total.
